# The contrasting N management of two oilseed rape genotypes reveals the mechanisms of proteolysis associated with leaf N remobilization and the respective contributions of leaves and stems to N storage and remobilization during seed filling

**DOI:** 10.1186/s12870-015-0437-1

**Published:** 2015-02-21

**Authors:** Alexandra Girondé, Philippe Etienne, Jacques Trouverie, Alain Bouchereau, Françoise Le Cahérec, Laurent Leport, Mathilde Orsel, Marie-Françoise Niogret, Nathalie Nesi, Deleu Carole, Fabienne Soulay, Céline Masclaux-Daubresse, Jean-Christophe Avice

**Affiliations:** Université de Caen Basse-Normandie, F-14032 Caen, France; UCBN, UMR INRA–UCBN 950 Ecophysiologie Végétale, Agronomie & Nutritions N.C.S., F-14032 Caen, France; INRA, UMR INRA–UCBN 950 Ecophysiologie Végétale, Agronomie & Nutritions N.C.S., F-14032 Caen, France; INRA, UMR 1349 Institut de Génétique, Environnement et Protection des Plantes, INRA, Agrocampus Ouest, Université de Rennes 1, F-35653 Le Rheu, France; UMR 1345 Institut de Recherche en Horticulture et Semences, SFR 4207 QUASAV, PRES L’UNAM, Université d’Angers, F-49045 Angers, France; UMR 1345 Institut de Recherche en Horticulture et Semences, AgroCampus-Ouest, F-49045 Angers, France; Département Adaptation des Plantes à l’Environnement, UMR 1318, INRA, Institut Jean-Pierre Bourgin, RD10, 78026 Versailles, Cedex France

**Keywords:** *Brassica napus*, Leaf senescence, N remobilization efficiency, N use efficiency, Proteolysis, Proteasome

## Abstract

**Background:**

Oilseed rape is the third largest oleaginous crop in the world but requires high levels of N fertilizer of which only 50% is recovered in seeds. This weak N use efficiency is associated with a low foliar N remobilization, leading to a significant return of N to the soil and a risk of pollution. Contrary to what is observed during senescence in the vegetative stages, N remobilization from stems and leaves is considered efficient during monocarpic senescence. However, the contribution of stems towards N management and the cellular mechanisms involved in foliar remobilization remain largely unknown. To reach this goal, the N fluxes at the whole plant level from bolting to mature seeds and the processes involved in leaf N remobilization and proteolysis were investigated in two contrasting genotypes (Aviso and Oase) cultivated under ample or restricted nitrate supply.

**Results:**

During seed filling in both N conditions, Oase efficiently allocated the N from uptake to seeds while Aviso favoured a better N remobilization from stems and leaves towards seeds. Nitrate restriction decreased seed yield and oil quality for both genotypes but Aviso had the best seed N filling. Under N limitation, Aviso had a better N remobilization from leaves to stems before the onset of seed filling. Afterwards, the higher N remobilization from stems and leaves of Aviso led to a higher final N amount in seeds. This high leaf N remobilization is associated with a better degradation/export of insoluble proteins, oligopeptides, nitrate and/or ammonia. By using an original method based on the determination of Rubisco degradation in the presence of inhibitors of proteases, efficient proteolysis associated with cysteine proteases and proteasome activities was identified as the mechanism of N remobilization.

**Conclusion:**

The results confirm the importance of foliar N remobilization after bolting to satisfy seed filling and highlight that an efficient proteolysis is mainly associated with (i) cysteine proteases and proteasome activities and (ii) a fine coordination between proteolysis and export mechanisms. In addition, the stem may act as transient storage organs in the case of an asynchronism between leaf N remobilization and N demand for seed filling.

**Electronic supplementary material:**

The online version of this article (doi:10.1186/s12870-015-0437-1) contains supplementary material, which is available to authorized users.

## Background

Over the last four decades, oilseed rape (*Brassica napus* L.) has become the third most widely grown oleaginous crop worldwide with a 2.4-fold increase in seed production between 1992 and 2012 [1]. This crop represents a major renewable resource for human food (oil), animal feed (meal) and numerous non-food uses (biofuel, lubricants, high added-value products derived from green chemistry). To increase the level of seed production with current genotypes, the use of N fertilizers has increased by 430% over the last forty years [2]. Despite the high capacity of mineral N absorption of oilseed rape [3], only 50% of N from fertilizer is recovered in seeds at harvest [4]. This low N Use Efficiency (NUE) is related to the loss of N by leaf drop (up to 100 kg.N.ha^−1^.year^−1^, [5]), reflecting the weak N Remobilization Efficiency (NRE) of oilseed rape. Therefore, in a context of imposed limitations on N-fertilizer inputs, improving NUE is becoming a priority in order to maintain/increase seed yield and decrease (i) the risk of water pollution by nitrate, (ii) the emission of greenhouse gases contributing to global warming, and (iii) the economic costs of oilseed rape crops.

NUE can be represented as two main components: the N Uptake Efficiency (NUpE) and the N Utilization Efficiency (NUtE), itself subdivided into two other components, N Assimilation Efficiency (NAE) and N Remobilization Efficiency (NRE) [6]. Even if N uptake of winter oilseed rape is considered to be efficient at vegetative stages [3], an N uptake that remains significant until flowering is associated with genotypes having high seed yield [7]. A mineral N input during seed filling increases seed yield of spring oilseed rape (*cv*. Aries, [8]) and recent studies on winter oilseed rape [9-13] have reported that a N uptake during reproductive stages appears to be a determinant trait for seed yield of winter oilseed rape, especially in restricted N supply. Nevertheless, in field conditions, the mineral N availability highly fluctuates during reproductive stages due to environmental factors, such as water deficit in the soil. To obtain a high NUE, the N coming from uptake has to be well managed by the plant. Consequently, an improvement of NUtE is also necessary to enhance NUE. Concerning the NAE, transgenic approaches have been targeted to enzymes involved in N metabolism such as nitrate reductase, nitrite reductase or alanine aminotransferase (AlaT) in various species such as *Arabidopsis* and tobacco, with more or less success (for review [14]). A promising result was obtained with oilseed rape plants overexpressing an AlaT, which need 40% less N fertilizer to reach similar yield as the wild type [14]. However, a study of 40 spring oilseed rape genotypes has suggested that NRE is a major determinant of NUtE [15] and an efficient assimilation needs to be correlated to an enhanced N remobilization to improve the seed N filling, which is essential to improve/maintain seed yield, in a context of N input reduction.

The N remobilization associated with leaf senescence is considered as crucial for oilseed rape yield [16]. During the vegetative stages, N is remobilized from the older leaves to the younger leaves along the axis of the plants *via* the sequential senescence, but senescent leaves can fall with a high level of residual N (up to 3.5% of dry matter) leading to a significant return of N to the soil [5,17]. By developing a modelling approach as a mean to identify potential methods for improving the NUE of oilseed rape, it was estimated that a 1% decrease in the residual N in fallen leaves (from 3.5% to 2.5% of dry matter), resulting from an enhanced foliar N remobilization, may increase seed yield by 5-10% [18]. After bolting, and especially during monocarpic senescence when the N is remobilized from vegetative organs to seeds, the low soil N availability during spring and at the beginning of summer [19] makes N remobilization crucial for seed yield and seed filling. During this period of development the main source organs are leaves, stems and pod walls [16,17,20,21]. During reproductive stages, the N amount in source leaves is highly remobilized (86% of N present at the beginning of flowering in field conditions) [12], leading to a low residual N in dead leaves (less than 2% of dry matter; [17]) and resulting in the reduction of the risk of N pollution. These results highlight an efficient N remobilization from source organs during monocarpic senescence, but also reveal variations for the residual N amount in stems compared with leaves of four winter oilseed rape genotypes [12]. In addition, a genotypic and N-supply interaction for N content in stems was found for 12 genotypes of spring canola [22] and the genotype with the best N remobilization was able to better remobilize N from stems and pod walls to seeds [23]. These results suggest that stems can also be important organ for improving seed N-filling, but the role of stems in N management at the whole plant level for winter oilseed rape remains largely unknown.

Although NRE seems to be a major lever for improving the NUE in oilseed rape, the cellular mechanisms associated with N remobilization from senescing leaves (proteolysis and the N export) remain largely an enigma. During leaf senescence, the degradation of proteins (the main form of N storage in leaves) into amino acids or peptides is performed by different classes of proteases. Studies on *Arabidopsis* reported the predominant involvement of cysteine and serine proteases (for review [24]), but also a role for the proteasome in the degradation of carbonylated proteins, which are accumulated during senescence [25]. The Ribulose-1,5-biphosphate carboxylase/oxygenase (Rubisco; EC 4.1.1.39) can represent up to 65% of the soluble proteins in C3 plants [26] and 20–30% of total leaf N [27-29]. Consequently, Rubisco is the major source of N for remobilization and its budget is very relevant for the plant. In winter oilseed rape, cysteine, aspartic, and metallo-proteases as well as the 26S proteasome are supposed to play an important role in foliar N remobilization during senescence in the vegetative stages [30,31] but there is no evidence concerning the proteolysis activities that are involved in leaf senescence and degradation of Rubisco after the bolting stage.

Among the enzymes involved in the conversion of amino acids into transportable forms, a recent study highlighted the importance of asparagine synthetase (AS; EC 6.3.5.4) in N remobilization during senescence in *Arabidopsis* [32]. In addition, the glutamine synthetase (GS; EC 6.3.1.2) catalyses the assimilation of ammonium into glutamate to synthesize glutamine. Glutamine was found at high levels in phloem sap of oilseed rape (*cv*. DSV15 and Duplo; [33]) and consequently, GSs are supposed to be largely involved in N remobilization processes during senescence in winter wheat [34] and maize [35], especially the cytosolic form (GS1) in the case of oilseed rape [36,37]. In parallel, glutamate dehydrogenase (GDH; EC 1.4.1.2; [38]) may catalyse a glutamate deamination [39,40] which provides ammonium for the GS activity in senescing leaves. In addition, in case of high levels of ammonium, GDH can incorporate ammonium on α-ketoglutarate to produce glutamate [41]. A significant quantity of glutamate has been found in phloem sap of oilseed rape [33,42] and an increase of both GDH activities in sliced leaves of oilseed rape was associated with a decrease in soluble proteins and ammonium [43], suggesting an important role for GDH in foliar N remobilization. The phloem loading of the resulting amino acids is regarded as not limiting in oilseed rape at the vegetative stages [44]. The efficient export of amino acids and the involvement of GS1 and GDH in N remobilization need to be confirmed in leaves as they senesce after bolting because they are proposed to have an efficient N remobilization.

The aim of this study was to identify the physiological traits involved in the high NUE and N remobilization in restricted N supply conditions, at whole plant (from bolting to mature seeds) and cellular levels (from bolting to flowering stages). To reach this goal, two genotypes (Aviso and Oase), known to have different strategies following a nitrate limitation at the vegetative stage [45], were selected. Long term pulse-chase ^15^ N-labelling was performed to precisely define the endogenous and exogenous N fluxes at the whole plant level between the bolting and mature seed stages, under restricted and ample nitrate supply. A specific ^15^ N-labelling was also used in order to determine a relevant estimation of NUE and its different components (NUpE, NUtE and NRE). This study (i) highlights the role of leaves and stems in the remobilization of N towards seeds and (ii) allows the identification of the physiological traits associated with a high NUE in response to restricted N supply. Moreover, a study of foliar N remobilization in source leaves was performed through the analysis of foliar N compounds, the GS and GDH activities, and the development of a new method to study proteolytic activities, using endogenous Rubisco as a substrate, in order to determine the involvement of different classes of proteases in the N remobilization which occurs during this growth stage.

## Results

### Impact of N limitation on growth, yield components, seed and oil composition

**Figure 1:**
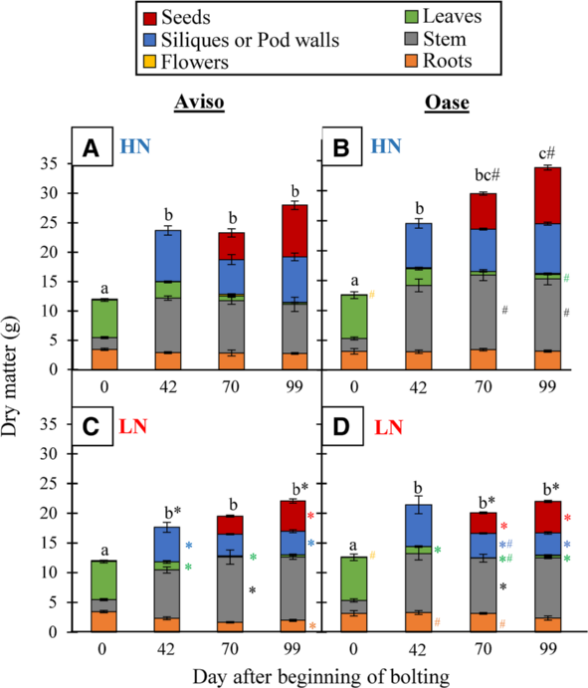
**Kinetics of dry matter (DM) of Aviso and Oase under ample or low N supply.** The plants were supplied with ample (HN, 3.75 mM) or low (LN, 0.375 mM) concentrations of nitrate. Dry matter is expressed in g per plant for Aviso **(A,** **C)** and Oase **(B,** **D)** at 0 (early bolting), 42 (pod formation), 70 (start of seed filling) and 99 (mature seeds) days after the beginning of bolting (D0). Seeds and pod walls were grouped and called siliques at D42 and they were separated from D70 onwards. Data are indicated as the mean value ± standard error (vertical bars). Different letters (a, b, c) indicate that the total dry matter is significantly different between two dates. Hashes represent significant differences between genotypes in HN or LN conditions and the asterisks represent significant differences between N treatments (*n* = 4 plants; *p* < 0.05).

When plants were well supplied with nitrate (HN), the total DM (Figure 1A, B and Additional file 1) was higher for Oase (34 g) compared with Aviso (28 g) during seed filling (D70 to D99), mainly due to differences in the stem DM (12.1 g for Oase *vs* 8.4 g for Aviso). Under our experimental conditions, the occurrence of the different stages of development was not significantly different between both genotypes or between both N treatments for a given genotype. In low nitrate (LN) conditions, no difference in the total DM was observed between genotypes (Figure 1C, D and Additional file 1). As expected, compared with HN, DM was reduced in leaves from D42, in stems at D70 and in pod walls at D99. In addition, DM declined significantly in siliques at D42 and increased in stems at D70 for Aviso while DM of siliques and stems declined at D70 for Oase. The root DM at D99 was lower in Aviso under LN compared with HN. Under LN supply, a similar decrease in seed production was observed for both genotypes (−41.8% for Aviso, −42.8% for Oase; Figure 1C and D).

In HN plants, the yield components (C:N ratio, seed N amount, N Harvest Index (NHI); Table 1) as well as the seed composition (proteins and oil in % of dry matter (DM); Table 1) were similar for both genotypes, except for the percentage of omega-3 (C18:3) and omega-6 (C18:2) precursors, which were higher for Aviso than Oase. However, the C18:2/C18:3 ratio was similar for both genotypes (1.9). As expected, an N limitation led to a strong decrease in the N amount of seeds (−53% in Aviso, −61% in Oase; Table 1). Compared with HN conditions, NHI significantly increased in Oase and tended to increase in Aviso (*p* = 0.06) in response to LN treatment (Table 1). Seeds of Oase LN plants had a higher C:N ratio associated with a higher oil percentage, which was to the detriment of proteins (20% proteins in DM *vs* 26% for Aviso). For oil composition, an increase of oleic acid (C18:1) proportion was observed under N limitation for both genotypes (Table 1). While C18:3 and C18:2 percentages did not differ under both N conditions for Aviso, an increase in C18:2 (+2%) and in the C18:2/C18:3 ratio (2.3) occurred under LN for Oase. The proportion of erucic acid also increased under LN conditions for Aviso (8% of oil).

**Table 1:** **Seed composition and nitrogen harvest index of Aviso and Oase under ample or low N supply**

		**Aviso**		**Oase**	
		**HN**	**LN**	**HN**	**LN**
**Yield components**	C:N ratio	11.31 ± 0.19	14.29 ± 1,01^*^	11.19 ± 0.25	18.36 ± 0.70^**#^
	Seed N amount (mg)	421.48 ± 40.08	194.36 ± 8.05^**^	432.84 ± 5.93	168.22 ± 7.77^**#^
	N Harvesting Index (NHI)	63.69 ± 3.33	72.05 ± 2.14	65.01 ± 1.24	73.97 ± 1.68^*^
**Seed composition**	Proteins (%)	32.20 ± 0.39	25.83 ± 1.20^*^	31.68 ± 1.87	19.48 ± 0.77^*#^
	Fatty acids (%)	33.99 ± 1.31	38.32 ± 1.45^*^	30.10 ± 4.27	44.37 ± 1.75^*#^
	C18:1 (oleic; %)	53.13 ± 0.46	55.86 ± 0.8	58.76 ± 3.13	64.61 ± 1.08^*#^
	C18:2 (linolenic ω6; %)	21.91 ± 0.34	21.43 ± 0.33	15.34 ± 0.40^#^	17.29 ± 0.29^*#^
	C18:3 (linoleic ω3; %)	11.53 ± 0.19	10.91 ± .022	8.05 ± 0.59^#^	7.56 ± 0.29^#^
	C22:1 (erucic; %)	2.13 ± 0.13	8.26 ± 0.83^*^	3.57 ± 2.39	4.28 ± 2.19^#^

The plants were supplied with an ample (HN, 3.75 mM) or low (LN, 0.375 mM) nitrate concentration. The N amount in seed is estimated in milligrams. C: N ratio: ratio C: N in seeds; NHI: N Harvesting Index (mg N in seeds per mg N in shoot). The seed composition (percentage of proteins and fatty acids) and the oil composition (percentage in oleic, linolenic, linoleic and erucic acids) was determined by NIRS method. Asterisks represent significant differences between N treatments and hashes represent significant differences between the two genotypes (*n* = 4 plants; #,*= *p* < 0.05; **= *p* < 0.01).

### Effects of nitrate limitation on N amount and NUE components

**Figure 2:**
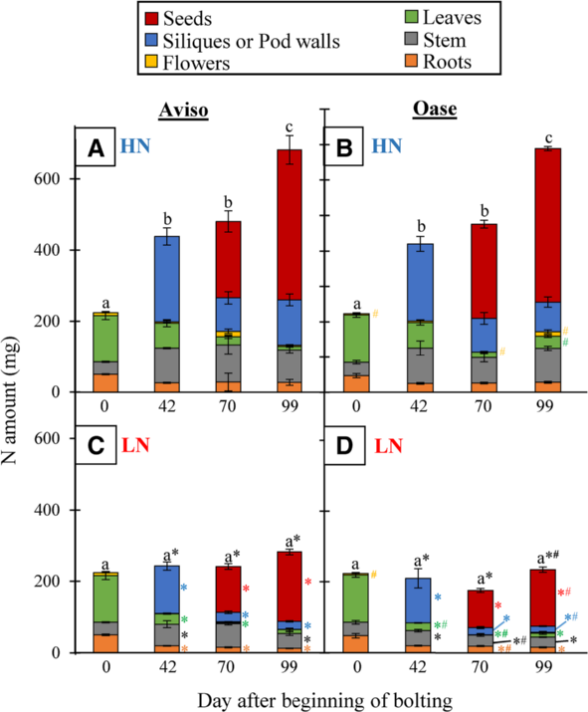
**Kinetics of the N amount in Aviso and Oase under ample or low N supply.** The plants were supplied with ample (HN, 3.75 mM) or low (LN, 0.375 mM) concentrations of nitrate. The N amount is expressed in mg per plant for Aviso **(A,** **C)** and Oase **(B,** **D)** at 0 (early bolting), 42 (pod formation), 70 (start of seed filling) and 99 (mature seeds) days after the beginning of bolting (D0). Seeds and pod walls were grouped and called siliques at D42 and they were separated from D70 onwards. Data are indicated as the mean value ± standard error (vertical bars). Different letters (a, b, c) indicate that the total N amount is significantly different between two dates. Hashes represent significant differences between genotypes in HN or LN conditions and the asterisks represent significant differences between N treatments (*n* = 4 plants; *p* < 0.05).

In HN plants, the total N amount did not differ between the genotypes (Figure 2A, B and Additional file 2). However, the N amount in leaves and flowers at the final stage of development was significantly higher for Oase than for Aviso. As expected, the total N amount was reduced for both genotypes in LN conditions (Figure 2C, D and Additional file 2), resulting from a decline in the N amount in nearly all organs from D42. Exceptions were flowers present at any time during the experiment, roots at D42 for Oase, stems at D70 and leaves at D99 for Aviso, which all showed a similar N amount in both N conditions. In LN plants, the final N amount in seeds and pod walls of Oase was lower than in Aviso (D99).

The N use efficiency (NUE), N utilization efficiency (NUtE), and N remobilization efficiency (NRE) were negatively correlated to N supply while the N uptake efficiency (NUpE) was positively correlated to N supply (Table 2). The NUE was higher for Oase (2.29) than for Aviso (1.98) in HN conditions while no differences were observed in the LN treatment (2.86 for Oase and 3 for Aviso). The NUtE increased in response to LN supply but no differences were observed between the genotypes, whatever the N supply. A strong genotype/treatment interaction effect was observed for NUpE between D70 and D99. In HN conditions, the NUpE of Oase was around 81%, suggesting that the N distributed to seeds during this period is mainly provided by the N that is newly taken up by the roots. The NUpE of Oase is about 2-fold higher than Aviso in both N conditions. The global NRE, *i.e.* the NRE calculated on the whole growing cycle (D0-D99; Table 2), reached about 55-60% for both genotypes, indicating that a large part of the N stored in source organs at the bolting stage (D0) is remobilized during the experiment. When NRE was calculated stepwise (*i.e.* between D0-D42, D42-D70 and D70-D99), a regular decrease occurred throughout the steps, whatever the treatment and the genotype. In HN plants, NRE was 1.2-fold higher in Oase than Aviso between D42 and D70 while it was reduced by about 50% after D70 (32.7 for Aviso *vs* 14.9 for Oase). In response to LN supply, global NRE increases for both genotypes, but a genotypic effect was highlighted between D70 and D99, where the NRE of Aviso was 1.4-fold higher than Oase (Table 2). However, no genotype/treatment interaction was observed for NRE.

**Table 2:** **NUE, NUtE, NUpE and NRE of Aviso and Oase under ample or low N supply**

		**Genotypes (G)**				**Source of variation**			
		**Aviso**		**Oase**		**G**	**N**		**G x N**
**N treatment (N)**		**HN**	**LN**	**HN**	**LN**	**F**_**G**_	**F**_**N**_	**r**	**F**_**GxN**_
NUE^a^		1.98 ± 0.06	3.00 ± 0.12^**^	2.29 ± 0.11^#^	2.86 ± 0.08^**^	1.048	89.81^***^	−0.90	7.13^*^
NUtE^b^		13.25 ± 0.66	20.27 ± 2.00^*^	13.67 ± 0.50	23.45 ± 0.55^*^	3.38	73.78^***^	−0.90	1.10
NUpE^c^ (%; D70-D99)		45.86 ± 8.10	32.00 ± 5.30	81.57 ± 4.25^#^	70.32 ± 7.88^#^	8.72*	60.81^***^	0.76	22.96^**^
NRE^d^ (%)	**Global**								
	0-99	59.12 ± 1.99	74.10 ± 3.22^**^	52.83 ± 2.03	76.08 ± 4.10^**^	0.70	55.28^***^	−0.89	2.59
	**Stepwise**								
	0-42	72.28 ± 3.48	86.62 ± 4.37^*^	77.59 ± 4.52	83.26 ± 4.16	0.07	7.74^*^	−0.60	1.45
	42-70	51.17 ± 1.46	77.80 ± 4.16^**^	59.90 ± 2.92^#^	75.65 ± 11.29	0.37	15.40^**^	−0.73	1.01
	70-99	32.70 ± 2.75	39.96 ± 2.19	14.91 ± 1.13^#^	27.70 ± 1.75^**#^	71.99^***^	31.99^***^	−0.52	2.43

The plants were supplied with an ample (HN, 3.75 mM) or low (LN, 0.375 mM) nitrate concentration. D0 corresponds to the early bolting stage, D42 to pod formation, D70 to seed filling and D99 to mature seeds. The main source of variation is deduced from an ANOVA test where genotypes, N treatment, and genotype x N treatment interaction were tested. The resulting F values are presented below. The r values correspond to the correlation between N supply and NUE, NUtE, NUpE and NRE, respectively.

^a^:NUE (Nitrogen Use Efficiency) corresponds to the mg of N in seeds per mg of the N amount in the whole plant relative to the seed DM (g) produced per g of total DM (including roots, pod walls and seeds).

^b^:NUtE (Nitrogen Utilization Efficiency) corresponds to the g of seed DM produced per mg of N in shoots (including pod walls and seeds).

^c^:NUpE (Nitrogen Uptake Efficiency) is estimated as the percentage of N derived from uptake in the total N (from remobilization and uptake) distributed to seeds between D70 and D99 (% of N distributed to seeds that derived from uptake).

^d^:NRE (Nitrogen Remobilization Efficiency) is determined as the percentage of N stored in all source organs which is remobilized (the total remobilized N amount relative to the total N amount in all sources) between D0 (bolting stage) and final harvest (D99) or between D0 and D42, D42 and D70 and D70 and D99.

Asterisks represent significant differences between N treatments and hashes represent significant differences between the two genotypes (*n* = 4 plants; #,*= *p* < 0.05; **= *p* < 0.01; ***= *p* < 0.001).

### N fluxes at the whole plant level in HN conditions

**Figure 3:**
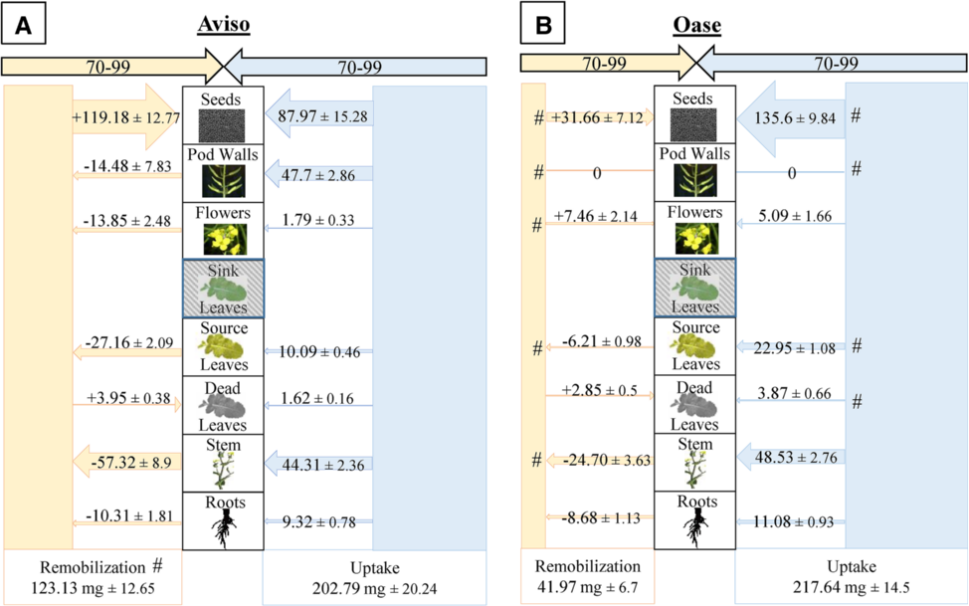
**N fluxes in Aviso (A) and Oase (B) in HN conditions between D70 and D99.** The plants were supplied with an ample concentration of nitrate (HN, 3.75 mM of nitrate). D70 corresponds to the start of seed filling and D99 to the mature seed stage. Fluxes of N from remobilization or uptake in the different organs are expressed as mg of N remobilized or taken up, respectively. A shaded box means that the organ was not present during these growing stages. For fluxes of N remobilization, the N amount is indicated with a minus sign (−) when N is remobilized from a source organ, or it is indicated with a plus sign (+) when remobilized N is redistributed towards a sink organ. Data are indicated as the mean value ± standard error. Hashes represent significant differences between genotypes (*n* = 4 plants; *p* < 0.05).

The ^15^ N labelling method used in this study gave the opportunity to determine precisely the N fluxes at the whole plant level (remobilization and uptake) between D0-D42, D42-D70 and D70-D99. Due to the fact that genotype and N treatment effects were observed for NRE solely between D70 and D99 in HN plants (Table 2), only the N fluxes between these two growing stages are given in Figure 3 (for other growing stages, N fluxes are given in Additional files 3 and 4). In HN conditions, N remobilization was around 3-fold higher in Aviso (Figure 3A) than Oase (Figure 3B). For both genotypes, the stem was the main source organ: 47% (*i.e.* 57.32 mg N) and 59% (*i.e.* 24.70 mg N) of the total N remobilized in Aviso and Oase, respectively. For Aviso, the other source organs were the leaves, pod walls, flowers and roots while for Oase the source organs were leaves and roots. For Oase, the N amount remobilized from leaves was 4.4-fold lower than for Aviso. The N remobilized from source organs was mainly distributed to the seeds with a significantly greater amount in Aviso (119.18 mg N) than Oase (31.66 mg N). Contrary to Aviso, the flowers of Oase are sink organs (7.46 mg N) for remobilized N. There was no significant difference in the amount of N uptake between genotypes (202.8 mg N for Aviso and 217.6 mg N for Oase; Figure 3). However, the allocation of N towards seeds is more important for Oase (135.6 mg N) than for Aviso (87.97 mg N). Despite this higher allocation of N towards seeds in Oase, the total N distributed to Oase seeds was not the same as in Aviso (−40 mg N for Oase compared with Aviso; Figure 3).

### N fluxes at the whole plant level in response to nitrate limitation

**Figure 4:**
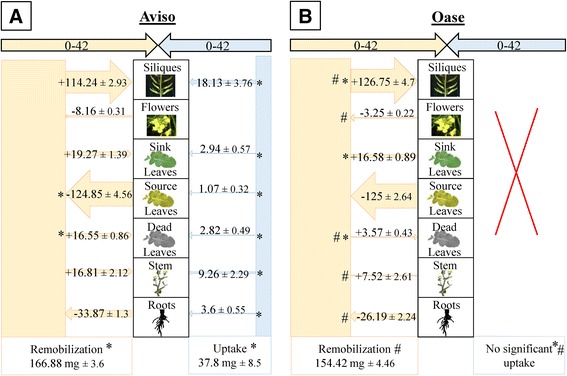
**N fluxes in Aviso (A) and Oase (B) in LN conditions between D0 and D42.** The plants were supplied with a low concentration of nitrate (LN, 0.375 mM of nitrate). D0 corresponds to early bolting and D42 to pod formation. Fluxes of N from remobilization or uptake in the different organs are expressed as mg of N remobilized or taken up, respectively. For fluxes of N remobilization, the N amount is indicated with a minus sign (−) when N is remobilized from a source organ, or it is indicated with a plus sign (+) when remobilized N is redistributed towards a sink organ. Data are indicated as the mean value ± standard error. Asterisks represent significant differences between treatments and hashes represent significant differences between genotypes (*n* = 4 plants; *p* < 0.05).

Between D0 and D42, N remobilization increased for Aviso in response to LN treatment (+20.6 mg N; Figure 4A) compared with HN (Additional file 3A), due to a larger N remobilization from source leaves (+18 mg N). This remobilized N was mainly redistributed towards siliques, allowing a similar amount of N to be redistributed as HN plants (around 110 mg N). In contrast to Aviso, Oase showed a similar total amount of remobilized N in both N conditions (154.4 mg and 150.6 mg N in LN and HN conditions, respectively; Figure 4B and Additional file 3B). However, compared with HN, the redistribution of this remobilized N to siliques was higher in Oase LN plants (+28 mg N) due to a lower loss *via* dead leaves and a lower redistribution to sink leaves (Figure 4B). The N remobilization in Oase was lower than Aviso (−12 mg N) due to a lower contribution of roots and flowers. As expected, in LN plants the N uptake strongly decreased for Aviso (only 37.82 mg N) and was not detected for Oase (Figure 4). In Aviso LN plants, the main sink organs for the N uptake were siliques, leaves and stems (Figure 4A). Thanks to the N uptake in Aviso, which supplemented the N remobilization, the total N amount distributed to siliques was similar in both genotypes (around 130 mg N; Figure 4).

**Figure 5:**
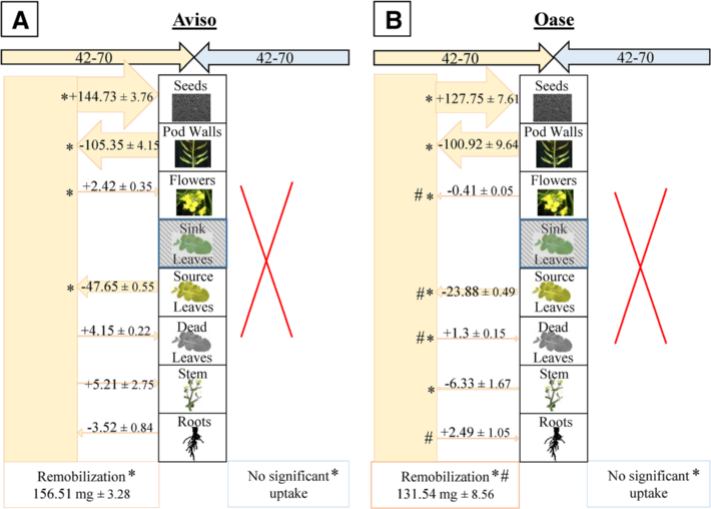
**N fluxes in Aviso (A) and Oase (B) in LN conditions between D42 and D70.** The plants were supplied with a low concentration of nitrate (LN, 0.375 mM of nitrate). D42 corresponds to pod formation and D70 to the start of seed filling. Fluxes of N from remobilization or uptake in the different organs are expressed as mg of N remobilized or taken up, respectively. A shaded box means that the organ was not present during these growing stages. For fluxes of N remobilization, the N amount is indicated with a minus sign (−) when N is remobilized from a source organ, or it is indicated with a plus sign (+) when remobilized N is redistributed towards a sink organ. Data are indicated as the mean value ± standard error. Asterisks represent significant differences between treatments and hashes represent significant differences between genotypes (*n* = 4 plants; *p* < 0.05).

Between D42 and D70, no N uptake was detectable and the total remobilized N amount decreased for both genotypes under N limitation (Figure 5) contrary to HN plants (Additional file 4). This was mainly related to lower contributions of leaves and pod walls, leading to a lower N redistribution to seeds. The N remobilization was 1.2-fold higher for Aviso than Oase (Figure 5), mainly due to a 2-fold higher N remobilization from leaves. Nevertheless, the N redistribution to seeds was not significantly different between the genotypes (144.73 mg N for Aviso and 127.75 mg N for Oase). These results can be explained by the fact that flowers and stems are sink organs for Aviso contrary to Oase, and a higher N loss by dead leaves occurs for Aviso.

**Figure 6:**
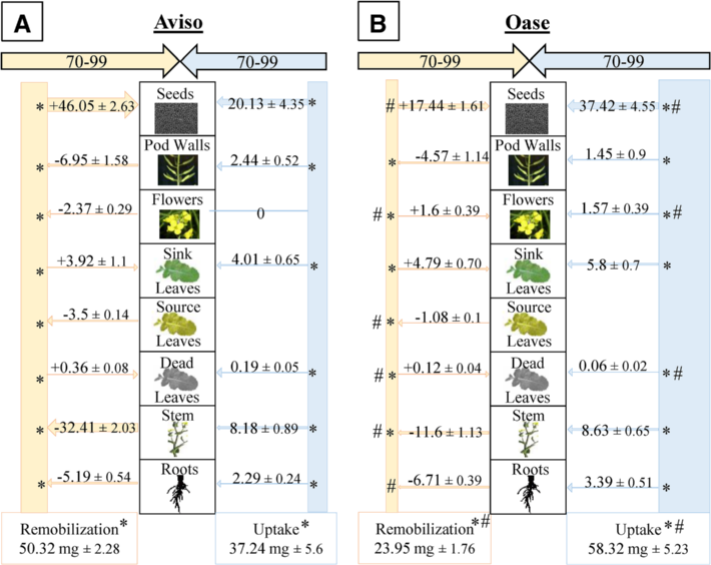
**N fluxes in Aviso (A) and Oase (B) in LN conditions between D70 and D99.** The plants were supplied with a low concentration of nitrate (LN, 0.375 mM of nitrate). D70 corresponds to the start of seed filling and D99 to mature seeds. Fluxes of N from remobilization or uptake in the different organs are expressed as mg N remobilized or taken up, respectively. For fluxes of N remobilization, the N amount is indicated with a minus sign (−) when N is remobilized from a source organ, or it is indicated with a plus sign (+) when remobilized N is redistributed towards a sink organ. Data are indicated as the mean value ± standard error (vertical bars). Asterisks represent significant differences between treatments and hashes represent significant differences between genotypes (*n* = 4 plants; *p* < 0.05).

Between D70 and D99, the N remobilization from all source organs was low for both genotypes (Figure 6), leading to a lower total N remobilization in LN compared with HN plants (−59% for Aviso and −43% for Oase; Figure 3). Consequently, a lower N amount was redistributed to seeds (−73.13 mg N for Aviso and −14.22 mg N for Oase; Figure 6). Under LN conditions, Aviso had a higher amount of total remobilized N than Oase (+27 mg N) resulting in a higher redistribution of remobilized N to seeds (+29 mg N). This was related to a higher remobilization from stems (+21.81 mg N) and source leaves (+2.42 mg N) for Aviso compared with Oase. The flowers were sink organs for remobilized N in Oase LN plants (Figure 6B) in contrast to Aviso LN plants. It is noteworthy that contrary to the HN treatment, sink leaves were present for both genotypes (Figure 6). Unlike the previous period (D42-D70, Figure 5), a significant N uptake occurred in both genotypes under LN conditions (Figure 6). The N uptake and allocation of N taken up into seeds were respectively 1.57- and 1.86-fold higher for Oase than for Aviso. Nevertheless, the total N amount distributed to the seeds of Oase LN plants (*i.e.* from N uptake and N remobilization, 54.8 mg N) remained lower than the total N amount distributed to seeds of Aviso LN plants (66.2 mg N, Figure 6).

### Impacts of nitrate limitation on chlorophyll levels, N compounds and amino acid metabolism during N remobilization in a selected source leaf

**Figure 7:**
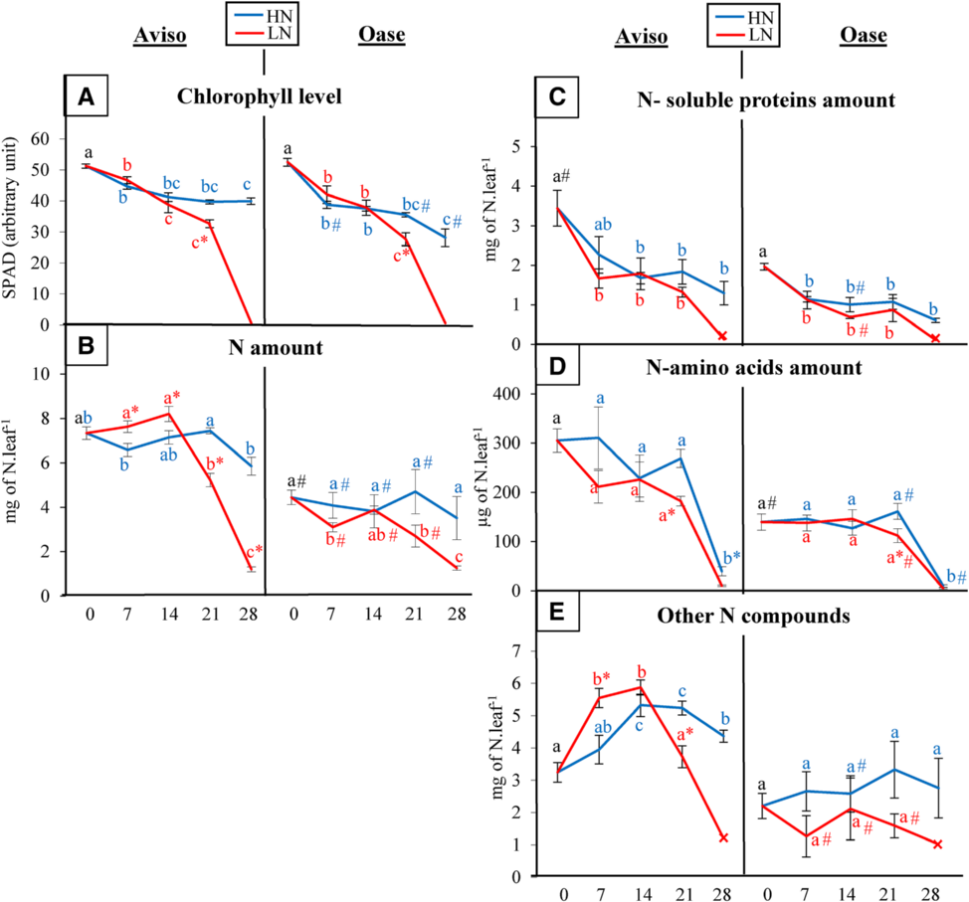
**Changes in chlorophyll level, total N, N-soluble proteins, N-amino acids and other N compounds in a source leaf.** Plants of Aviso and Oase were supplied with ample (HN, 3.75 mM) or low (LN, 0.375 mM) nitrate concentrations. These data were obtained on a selected “source leaf”, determined as mature at D0 (early bolting) and becoming senescent during the experiment. The chlorophyll amount (**A**; SPAD value) is expressed in an arbitrary unit. The amount of total N **(B)**, soluble proteins **(C)**, amino acids **(D)** and other N compounds **(E)** are expressed as mg of N per leaf for each fraction. The fraction of other N compounds that mainly corresponded to insoluble proteins, oligopeptides and ammonia, was determined as follows: mg of total N amount - (mg of N-soluble proteins + mg of N-amino acids). All these data were quantified at 0, 7 (bolting stage), 14 (flower buds raised above the youngest leaves), 21 (first petals visible, but flower buds still closed) and 28 (flowering) days after the beginning of bolting (D0). Concerning the soluble proteins and the other N compounds, only one biological replicate remained at D28, and its value is indicated by a cross (x). Data are indicated as the mean value ± standard error (vertical bars). Letters a, b and c represent differences in kinetics, asterisks indicate significant differences between treatments and hashes represent significant differences between genotypes (*n* = 4 plants; *p* < 0.05).

In order to study the remobilization at the foliar level in response to N limitation applied at the bolting stage (D0), selected mature leaves undergoing senescence during the experiment (called “source leaves”) were analysed in detail over 28 days. The leaf biomass (at D0), leaf area (from D0 to D28; Additional file 5) and chlorophyll content (at D0; Figure 7A) in these selected source leaves were not significantly different between Aviso and Oase, meaning that the leaf initial status was similar between both genotypes. Consequently, it was possible to compare the processes involved in the N remobilization of source leaves of both genotypes. In HN conditions, the chlorophyll content decreased at D7 and remained constant for Aviso until D28, while it decreased all along the experiment for Oase. In response to LN treatment, a decrease in chlorophyll content was observed from D21 for both genotypes (Figure 7A). In HN conditions, the N amount in the source leaf was 2-fold higher for Aviso than for Oase and remained nearly constant during the 28 days for both genotypes (Figure 7B). In response to LN conditions, the leaf N amount decreased significantly from D21 in Aviso alone, resulting in a decline of 83% (−6.13 mg N) between D0 and D28 (Figure 7B).

Considering the different N fractions, it is noteworthy that whatever the genotype, a similar pattern of soluble protein degradation (strong decrease at D7) was observed for both N conditions. However, the only available biological replicate at D28 (*n* = 1) suggested a lower amount of soluble proteins at D28 in LN plants compared with HN for both genotypes (Figure 7C). Even though the level of free amino acids was 1.7-fold higher in Aviso than in Oase, it decreased strongly in both genotypes at D28 in HN conditions (Figure 7D). In LN conditions, a lower amount of N-amino acids was observed for both genotypes at D21 compared with the HN treatment. Surprisingly, the fraction corresponding to the other N compounds increased in Aviso between D0 and D14 in both N conditions (+64% and +80% in HN and LN, respectively; Figure 7E). Thereafter, the fraction of other N compounds in the source leaf of Aviso remained almost stable in HN plants while it decreased strongly in LN plants (−82% between D14 and D28). Concerning Oase, the other N compounds remained low and constant during all the experiments, whatever the N supply.

**Figure 8:**
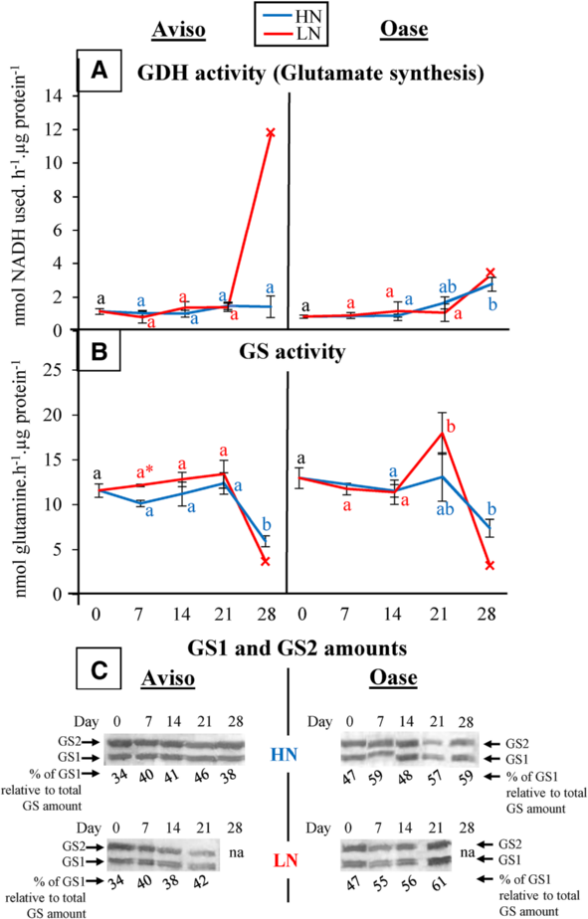
**Changes in glutamate dehydrogenase activity, glutamine synthetase activity and their amounts (GS1, GS2) in a source leaf.** Plants were supplied with ample (HN, 3.75 mM) or low (LN, 0.375 mM) nitrate concentrations. These data were obtained from a selected ‘source leaf’, determined as mature at D0 (early bolting) and becoming senescent during the experiment. The activity of glutamate dehydrogenase (GDH; **A**) was quantified as the synthesis of glutamate and is expressed as nmol of NADH used.h^−1^.μg^−1^ proteins. The activity of glutamine synthetase (GS; **B**) was determined by the nmol of glutamine produced.h^−1^.μg^−1^ proteins. The GS1 and GS2 amounts **(C)** were quantified after western blotting with specific antibodies and the percentage of GS1 among the total GS amount was estimated. Data were observed at 0, 7 (early bolting), 14 (flower buds raised above the youngest leaves), 21 (first petals visible, but flower buds still closed) and 28 (flowering) days after the beginning of bolting (D0). Only one biological replicate remained at D28, and its value is indicated by a cross (x). In panels A and B, data are indicated as the mean value ± SE (vertical bars). Letters a, b and c represent differences in kinetics, asterisks mean significant differences between treatment and hashes represent significant differences between genotypes (*n* = 4 plants; *p* < 0.05).

The activities of glutamate dehydrogenase (GDH) and glutamine synthetase (GS), involved in the metabolism and remobilization of amino acids during senescence, were similar and constant in both N conditions for Aviso, except for a 50% decrease in GS activity at D28 in both N conditions and a putative 6-fold increase (*n* = 1) of GDH activity at D28 in LN conditions (Figure 8A and B). Concerning Oase, the GDH activity remained low until D21 and increased by 1.5-fold at D28 in both N conditions (Figure 8A). In HN plants, the GS activity remained constant until D21 and decreased by 43% at D28 (*n* = 1). In LN plants, the GS activity increased (1.5-fold) at D21 and putatively decreased at D28 (*n* = 1, Figure 8B). The immunoblots of cytosolic (GS1) and chloroplastic (GS2) glutamine synthetase (Figure 8C) revealed that the proportion of GS1 was not impacted by LN treatment in both genotypes, but it remained higher for Oase throughout the experiment (53% for Oase and 39% for Aviso on average).

### Proteolytic activities in the source leaves

**Figure 9:**
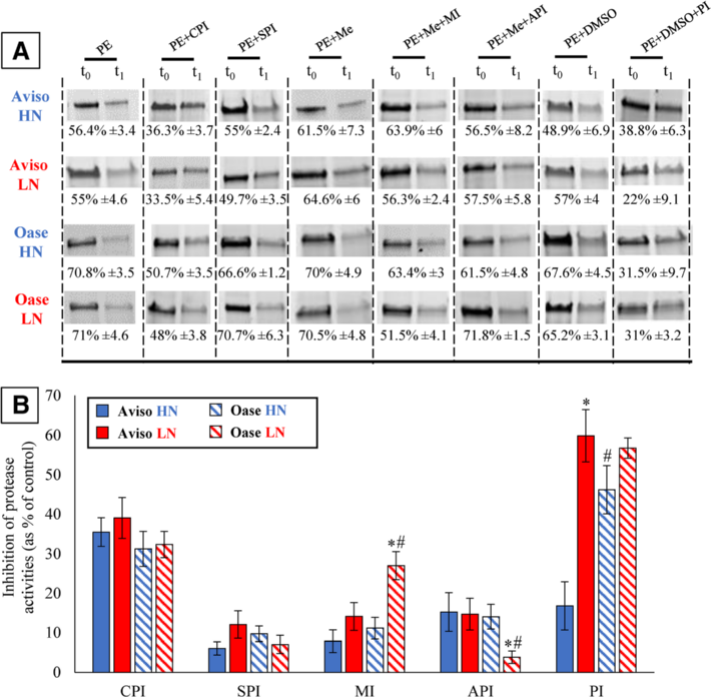
**Rubisco large subunit degradation in a source leaf with or without protease inhibitors (A) and the inhibition of the protease activities by protease inhibitors (B).** The Rubisco large subunit (LSU) in the soluble protein extract (PE) of the source leaf (7 days after bolting) is visualized on stain free SDS-PAGE and quantified for the four biological repetitions by Image Lab software (Bio-Rad) at (t_0_) and after 1 h of incubation at 37°C (t_1h_) without inhibitors (PE, control conditions) or with specific protease inhibitors: iodoacetamide (PE + CPI; cystein protease inhibitor), aprotinin (PE + SPI; serine protease inhibitor), methanol (PE + Me), methanol and 1–10 phenanthroline (PE + Me + MI; metalloprotease inhibitor), methanol and pepstatin A (PE + Me + API; aspartic protease inhibitor), DMSO (PE + DMSO) or DMSO and MG132 (PE + DMSO + PI; proteasome inhibitor). The most representative biological repetition is shown in panel A and the percentage of degradation (mean value ± SE, *n* = 4 plants) are indicated below. Panel B presents the inhibition of the protease activities by the proteases inhibitors (expressed as % of LSU degradation observed in control conditions (PE)). In panel B, data are indicated as the mean value ± SE. An asterisks means that the LSU degradation is significantly different between N treatment and # means a significant differences between genotypes (*n* = 4 plants; * or #= *p* < 0.05).

Different protease inhibitors (against cysteine, serine, aspartic or metallo- proteases and proteasome) were used in order to identify the class of proteases involved in the strong degradation of soluble proteins occurring in the source leaf at D7 (Figure 7C). Due to the fact that Rubisco represents a large proportion of the soluble proteins in leaves [26], the characterization of proteases was determined *via* the analysis of the degradation of the Rubisco large subunit (LSU, Figure 9A). In source leaf of HN plants (Figure 9B), the LSU proteolysis was strongly inhibited by iodoacetamide for Aviso and Oase (35.5 and 31.3% of inhibition, respectively) and by MG132 for Oase (46.22% of inhibition), suggesting that the proteolysis is mainly carried out by cysteine proteases for Aviso and by the proteasome and cysteine proteases for Oase. The contribution of cysteine and aspartic proteases to the LSU degradation in Aviso was the same in both N conditions, while a slight increase of serine proteases (from 6.1 to 12.1% of inhibition; *p* = 0.12) and metalloproteases (from 7.9 to 14.1% of inhibition; *p* = 0.17) and a significant increase of proteasome activity (60% of inhibition) were observed (Figure 9B) in response to the LN treatment. The contribution of proteasome, cysteine and serine proteases for Oase remained similar in both N conditions. Compared with HN plants, the contribution of aspartic proteases decreased (4% of inhibition) while the participation of metalloproteases increased (27% of inhibition) in Oase LN plants (Figure 9B).

## Discussion

### Evidence of two different N management strategies that lead to a similar seed yield under ample nitrate nutrition

The ^15^N-labelling method performed in this study allowed the distinction between endogenous and exogenous N fluxes and, therefore, the relevant determination of the involvement of N uptake and remobilization during seed filling. Despite no significant differences in seed yield (Figure 1), the two genotypes of winter oilseed rape investigated in the present work showed contrasting N management during seed filling under ample nitrate supply. In fact, even if both genotypes used exogenous and endogenous N for seed filling (Figure 3), Oase had the highest NUpE (82%) while Aviso had the highest NRE (32%) during seed filling (Table 2). Our data suggest that allocation of N taken up into seeds is more efficient in Oase than Aviso, while the processes of N remobilization from source organs towards seeds during the final reproductive phases is higher in Aviso. In fact, during seed filling, the total N remobilization was 3-fold higher for Aviso than for Oase, which was a consequence of a higher remobilization from leaves, stems and pod walls (Figure 3), leading to a higher amount of N redistributed towards seeds (Figure 2C and D). These results confirm that leaves, stems and pod walls are the main source organs for N remobilization after flowering [8,16,17,20,46].

### Under nitrate limitation, N management in Aviso is more efficient than Oase for seed filling

Despite an increase of NHI, a decrease of seed yield was observed for Oase and Aviso in response to nitrate limitation (LN treatment) applied at the early bolting stage (Figure 1, Table 1). The seed oil percentage increased in response to LN conditions for both genotypes (Table 1) but the nutritional quality of oil of both genotypes was affected: increase of (i) C18:2/C18:3 ratio for Oase and (ii) proportion of erucic acid for Aviso (Table 1). These results confirm the strong relationship between N supply and oil quality [47] and suggest a genotypic variability about the oil quality in response to N limitation.

As expected, in response to LN treatment, the N uptake was lower throughout the experiment (Figures 4, 5 and 6) and the total N amount in seeds (mg N in total seeds) decreased for both genotypes (Table 1), leading to a lower protein content in parallel to the increase in oil content [48-50]. Nevertheless, a significant N uptake occurred during seed filling despite the low N supply, especially in Oase (Figure 6), supporting the fact that there is a significant N uptake capacity after flowering in oilseed rape [16,17]. Interestingly, growth of new leaves was observed during seed filling in response to LN conditions (Figure 6). Even if these new organs represent a sink for N, new leaves may help maintaining photosynthetic capacities during seed filling and raise the potential seed yield by increasing the allocation of C to seeds [7,51]. In addition, photosynthetically active leaves could participate in the improvement of N uptake [8], as previously suggested for winter oilseed rape [52]. Indeed, the N uptake is associated with the availability of photoassimilates in the roots [53]. Consequently, the higher N uptake of Oase during seed filling (Figure 6) may be related to the significant higher C content observed under N limitation in Oase roots at D70 and D99 (+4% and +5% respectively compared with Aviso; data not shown).

As in HN conditions, N from uptake is mainly used for seed filling in Oase, but due to the low N supply, a lower N amount is distributed to seeds compared with Aviso (Figure 6). In addition, even though Oase had the highest N uptake during seed filling, Aviso had the highest total N uptake from the bolting to mature seeds stages (75 mg N) in comparison with Oase (58 mg N) (Figures 4, 5 and 6). This result agrees with the previous studies reporting that the genotypic variability of N uptake is mainly observed under restricted N supply [11,15]. However, because both genotypes have the same seed yield under LN conditions (Figure 1), the positive correlation between N uptake and seed yield demonstrated in previous works on oilseed rape [9,11,12,15] was not confirmed in our experimental conditions. The higher N amount in seeds of Aviso could be due to the fact that, as in HN treatment, endogenous N is mainly used for seed filling. Indeed, Aviso had a higher NRE during seed filling (Table 2), leading to a higher redistribution of the N remobilized from leaves and stems toward seeds in comparison with Oase (Figure 6). In order to better understand the importance of N remobilization from leaves and stems in Aviso with its superior NRE, these organs were studied in detail.

### Impact of leaf N remobilization and its associated cellular processes in coping with N limitation

As previously observed for oilseed rape *cv*. Capitol [17,20], leaves are the main source organs between bolting and early pod formation, whatever the genotype or the N supply (Figure 4 and Additional file 1). In fact, the maintenance of the amount of N distributed to siliques in Aviso under LN conditions is linked to an increase in N remobilization from source leaves (Figure 4A) associated with an increase in the number of dead leaves (Additional file 6). The N remobilization in leaves of Oase (Figures 4B and 7B) and the number of dead leaves (Additional file 6) were similar in both N conditions, suggesting a similar progression of senescence for this genotype whatever the N supply, which has never been highlighted previously.

The mechanisms involved in N remobilization were then investigated in a selected source leaf, becoming senescent between the bolting and the flowering stages. Proteins are the main form of N storage in source leaves, especially the soluble protein Rubisco [54], and for both genotypes a massive degradation of the soluble proteins occurs 7 days after bolting under both N conditions (Figure 7C). The aspartic and serine proteases seem to contribute at a low level to the proteolysis in both genotypes (Figure 9B, Additional file 7), contrary to previous results reported for *Arabidopsis* [24]. Our study reveals a higher involvement of metalloproteases during N remobilization in the source leaves of Oase in response to LN supply (Figure 9B). These results are consistent with previous proteomics studies which showed the induction of metalloproteases (FtsH) during leaf senescence at the vegetative stages of winter oilseed rape (*cv*. Capitol) in response to limitation/privation in nitrate [31]. Because FtsHs are involved in the proteolysis of D1 or Lhcb3 proteins localized in the thylakoid membrane [55], our results suggests that a nitrate limitation may lead to an improvement in the degradation of thylakoid-bound proteins.

Except for Aviso in HN conditions, the classes of proteases having the greatest involvement in the massive *in vitro* proteolysis observed 7 days after bolting are cysteine proteases and the proteasome system (Figure 9B). The importance of cysteine proteases in leaf proteolysis is in agreement with previous studies on winter oilseed rape demonstrating that the cysteine protease SAG12 is highly induced at the transcriptomic [56] and proteomic [31] levels during leaf senescence. The efficient N remobilization observed for Oase in both N conditions and for Aviso in LN treatment seems to be correlated to a high contribution of the proteasome in the proteolysis mechanisms (Figure 9B), which confirm the previous studies on oilseed rape [31] and *Arabidopsis* [24]. In addition, in our *in vitro* conditions, the proteasome seems to be able to degrade the LSU of Rubisco. However, in cell, the proteasome complex is involved in degradation of proteins previously tagged with ubiquitin and is mainly localized in the nucleus and cytosol (around pH 7.5, [57]). Some studies in *Arabidopsis* shown that Rubisco is degraded in acidic organelles such as lytic vesicles or vacuoles (around pH 5, [58-60]) and/or directly in the stroma (characterized by a pH of 7.5 and a low amount of ubiquitin [61]). Taken together, these studies suggest that Rubisco is probably not degraded *in planta* by the proteasome system. However, a chloroplastic E3 enzyme, involved in the selectivity of the ubiquitin-proteasome system during plastid formation was recently found in the outer membrane of chloroplasts [62]. In addition, the proteolysis by the proteasome can occur without the involvement of ubiquitin in animal cells [63], suggesting that could also happen in plant cells. Consequently, the involvement of the proteasome pathway in the degradation of chloroplastic proteins seems to be possible and should be investigated further.

The efficient degradation of soluble proteins observed in the senescing leaf of Aviso under both N conditions (Figure 7C) was not correlated with a concomitant decrease in the total N amount (Figure 7B). In our study, there was no transient accumulation of N in the amino acid fraction (Figure 7D), confirming that the export of amino acids was not limiting, as indicated in earlier results obtained for winter oilseed rape *cv.* Lirajet [44]. Consequently, despite the decline in N-soluble proteins, the stability of total N in senescing leaf was related to a transient accumulation of other N compounds (Figure 7E), including transmembrane proteins, peptides, nitrate and/or ammonia. In addition, the degradation and/or export of these other N compounds seem to be responsible of the higher N remobilization of the source leaf observed at D21 under LN conditions (Figure 7E). In Aviso LN plants, the high decrease of other N compounds at D21 is followed by an increase of the amination activity related to GDH (Figure 8A), which catalyses the incorporation of ammonium on α-ketogluratate to produce glutamate in case of accumulation of ammonium [64] released by the catabolism of proteins and peptides [65]. The better degradation of transmembrane proteins and peptides in LN conditions could lead to an ammonium accumulation and, therefore, may explain the increase of amination by the GDH in order to detoxify the cell and produce glutamate, which is found in phloem sap of oilseed rape [33]. If this hypothesis is true, the lower level of other N compounds observed in source leaf of Oase in both N conditions (Figure 7B) could be due to an efficient degradation of transmembrane proteins and/or peptides into amino acids. The amino acids are not accumulated in the source leaf (Figure 7D), suggesting that they are well exported towards the phloem vessels. This could be related to the fact that GS1, the cytosolic isoform of GS which is known to be involved in N remobilization processes [36], is present in higher proportions in the source leaf of Oase than in Aviso one (Figure 8C). These data suppose that an efficient degradation of transmembrane proteins and/or peptides and a rapid conversion of amino acids into their transportable forms (such as glutamate and glutamine) by enzymes like GDH or GS1 in order to enhance their exportation from the mesophyll cells towards the phloem is an important physiological trait for the optimization of NRE. These results have to be confirmed and other enzymes involved in the conversion of amino acids, such as asparagine synthetase in *Arabidopsis* [32], have to be investigated in oilseed rape leaves.

### Stems play a pivotal role in N management as a transient N storage organ

In LN supply, the highest N amount in seeds of Aviso compared with Oase is linked to a large N remobilization (3-fold higher) from the stem to seeds during seed filling (Figure 6). As previously observed [9], the highest N remobilization is not related to a lower residual N in the stem at D99 (Additional files 3 and 4), but to a significant higher distribution of N towards the stem before seed filling (Figures 4 and 5). Consequently, the increase of N storage capacity in stems before the onset of seed filling may significantly improve the N remobilization from stems towards seeds. The same conclusion was observed about leaves during seed filling. However, while leaves were crucial source organs before early silique formation, their participation remained low during seed filling (only 7 and 5% of total N remobilization for Aviso and Oase, respectively; Figure 6). A high N remobilization from leaves towards the stem was previously associated with a high NUE in spring oilseed rape [23]. Taken together, results suggest that the stem is a crucial buffering organ for transient N storage in order to compensate the delay between the large N remobilization from leaves and the sink N demand for seed filling.

## Conclusions

As previously observed under restricted N supply, the higher N uptake from bolting to flowering is associated with the most efficient genotype for seed N filling (Aviso in our study). Nevertheless, under field conditions, the mineral N availability strongly fluctuates and is usually low during seed filling, meaning that a higher N uptake could not be sufficient to significantly improve the NUE. The investigation of the contrasting N management in Aviso and Oase genotypes confirms that remobilization during monocarpic senescence is efficient for leaves and stems. Consequently, an efficient remobilization and utilization of N taken up and N stored before the onset of seed filling is probably one of the main physiological trait for attaining a high NUE in an agronomic context of mineral N restriction. Up to the early phase of seed filling, N is mainly stored in leaves, and therefore an efficient foliar N remobilization is important to limit N loss from dead leaves and improving seed N filling together with a limitation of pollution risk. Our findings suggest that an efficient foliar NRE is mainly associated to (i) a high proteolysis by the cysteine proteases and proteasome system and (ii) a strong coordination between proteolysis, the export/degradation of oligopeptides and the export of amino-acids (involving the enzymes GS and GDH). Future developments are needed to confirm the relevance of these classes of proteases before their potential use in a “candidate protein-strategy” to identify QTLs related to high NRE. Our study also highlights that the improvement of NUE and seed N filling under low mineral N availability is highly related to an efficient storage of the N remobilized from leaves into the stem. Thus, it is likely that the stem may act as a transient storage organ in case of asynchronism between the massive remobilization of N coming from source leaves and its further utilization by seeds. Such physiological traits (efficient leaf N remobilization, transient N storage in stems) may be particularly relevant to breeding programs aimed at identifying *Brassica napus* genotypes having high NUE in response to the fluctuation of nitrate availability and low N fertilizer inputs.

## Methods

### Greenhouse experimentation

Two genotypes of winter oilseed rape (*Brassica napus* L. *cv.* Aviso and Oase) were cultivated in greenhouse conditions (Additional file 5). Plantlets were supplied for the first 2 months with a 25% Hoagland solution (1.25 mM Ca(NO_3_)_2_ 4H_2_O; 1.25 mM KNO_3_; 0.5 mM MgSO_4;_ 0.25 mM KH_2_PO_4_; 0.2 mM EDTA-2NaFe-3H_2_O; 14 μM H_3_BO_3_; 5 μM MnSO_4_; 3 μM ZnSO_4_; 0.7 μM (NH_4_)_6_Mo_7_O_24_; 0.7 μM CuSO_4_; 0.1 μM CoCl_2_). Thereafter, plants were vernalized for 8 weeks under a long photoperiod and low temperature (day: 10 h, 10°C; night: 14 h, 4°C; 90 mL of 25% Hoagland solution per plant renewed twice a week). During pre-culture and vernalization, light was supplied by High Pressure Sodium Lamps (Philips, MASTER GreenPower T400W) with a PAR (Photosynthetically Active Radiation) of 400 μmol photon.s^−1^.m^−2^ at the top of the canopy. After vernalization, plants were transferred to a greenhouse in pots containing perlite and vermiculite (2:1, v/v; one plant per pot; 40 plants per square metre), and exposed to a thermoperiod of 20°C (day) and 15°C (night). To estimate the N fluxes at the whole plant level, long term pulse chase ^15^N-labelling (at 2% atom excess) was applied from the first days of germination until the early bolting stage (Day 0 of the experiment (D0) corresponding to GS31, a growth stage determined from the scale of BBCH (Biologische Bundesanstalt, Bundessortenamt und CHemische Industrie)). At D0, ^15^N-NO_3_ labelling was stopped and two N treatments with unlabelled-NO_3_ were applied until the final harvest stage (D99; corresponding to GS99 *i.e.* the final stage of development with mature seeds and the whole plant in senescence): high nitrate (HN, 3.75 mM) and low nitrate (LN, 0.375 mM). Each day the nutrient solution was supplied automatically in an increasing volume as a function of the growth stages: 90, 120, 150, and 180 mL per plant at the start of the bolting, visible bud, flowering, and seed maturation stages, respectively.

Plants were harvested at different growing stages: D0 (GS31), D7 (GS32: bolting stage), D14 (GS53: flower buds raised above the youngest leaves), D21 (GS59: first petals are visible), D28 (GS60: flowering), D42 (GS70: end of flowering), D70 (GS73: pod filling) and D99 (GS99: mature seeds, whole plant in senescence). Plants were separated into roots (including taproot and secondary roots), leaves, stem (including the ramifications), siliques, seeds and pod walls (when siliques were dehiscent). The dry matter of each organ was determined after freeze-drying. The thousand seed weight and the total seed number were deduced from the weight of one hundred seeds after 48 h at 30°C.

In order to analyse foliar N remobilization, leaves were classified into three groups (dead leaves, old + mature leaves and young leaves) on the basis of their nodal position, their leaf area was determined by a LI-COR 300 area meter (LI-COR, Lincoln, NE, USA) and their chlorophyll content measured by SPAD (Soil Plant Analysis Development; Minolta, SPAD-502 model). Throughout the experiment, dead leaves were counted and harvested. Moreover, at D0, a mature leaf that had become senescent during the experiment (*i.e.* a “source” leaf for N) was tagged and studied in order to characterize the processes associated with leaf N remobilization from bolting (D0) to flowering (D28) (see Additional file 5 for more details about the selection of these source leaves). One half of this mature leaf was freeze-dried, weighed for dry matter and ground to a fine powder for further biochemical analyses. The other half of the mature leaf was immediately frozen in liquid nitrogen and stored at −80°C until further protein and molecular analyses.

### Determination of oil content, fatty acid composition and protein contents in seeds

As previously described by Dubousset *et al.* [66], seed samples (about 3 g) were scanned on a monochromator Near Infra-Red System (NIRSystem model 6500, FOSS NIRSystem Inc, Silver Spring, MD, USA) equipped with the transport module, in the reflectance mode. The results were predicted from an external calibration established for oil content and composition of fatty acids (CRAW, Gembloux, Belgium). Three determinations were performed for each biological replicate.

### Total N and ^15^N quantification, whole plant N fluxes and determination of NUE components

The total N and ^15^N amounts in each organ were analysed by an elemental analyser (EA3000, EuroVector, Milan, Italy) linked to a continuous flow isotope mass spectrometer (IRMS, IsoPrime GV instruments, Manchester, UK). Due to the long term pulse chase labelling described below, the ^15^N amount is representative of the total N amount in every organ; the variation of the ^15^N amount allows estimation of the endogenous N fluxes related to N remobilization and the exogenous N flux coming from uptake. The determination of source or sink status of leaves or other organs was done on the basis of their ^15^N amount, where a sink organ is characterized by a gain of ^15^N and a source organ by a decrease in the ^15^N amount. The calculation of the whole plant N fluxes are described in detail in Salon *et al*. [67].

The N use efficiency (NUE) was calculated as:

$$ \mathrm{N}\mathrm{U}\mathrm{E} = \left({\mathrm{QN}}_{\mathrm{seed}}/\ {\mathrm{QN}}_{\mathrm{whole}\ \mathrm{plant}}\right)\ /\ \left({\mathrm{DM}}_{\mathrm{seed}}/\ {\mathrm{DM}}_{\mathrm{whole}\ \mathrm{plant}}\right) $$

where QN_seed_ corresponds to the N amount (mg) in seeds, QN_whole__plant_ to the N in the whole plant (including roots and seeds), DM_seed_ to the seed dry matter (g) and DM_whole plant_ refers to the whole plant dry matter (g) observed at the final stage (including roots and seeds; GS99).

The N utilization efficiency (NUtE) corresponds to the seed dry matter (DM_seed_) production per g of N in shoots (QN_shoot_) and was estimated by the following calculation:

$$ \mathrm{NUtE} = {\mathrm{DM}}_{\mathrm{seed}}/{\mathrm{QN}}_{\mathrm{shoot}} $$

Based on the calculation of endogenous and exogenous N fluxes, the N remobilization efficiency (NRE; total remobilized N amount relative to the total N stored in source organs) and N uptake efficiency (NUpE; N amount allocated to seeds from uptake relative to total N distributed to seeds) were defined as follows (as a percentage):

$$ \mathrm{N}\mathrm{R}\mathrm{E} = \varSigma {\mathrm{QN}}_{\mathrm{remobilization}}*100\ /\ \left(\varSigma {\mathrm{QN}}_{\mathrm{source}}t + \varSigma {\mathrm{QN}}_{\mathrm{uptake}}\right) $$

$$ \mathrm{NUpE} = {\mathrm{QNS}}_{\mathrm{uptake}}*\ 100\ /\ {\mathrm{QNS}}_{\mathrm{distributed}} $$

where ΣQN_remobilization_ corresponds to the total N amount remobilized between *t* and *t +* Δ*t*, ΣQN_source_*t* is the N amount in all source organs at *t* and ΣQN_uptake_ corresponds to the amount of N taken up that is allocated to all source organs between *t* and *t +* Δ*t*. The QNS_uptake_ corresponds to the N amount allocated to seeds from the uptake and the QNS_distributed_ to the total N amount distributed to seeds (from uptake and remobilization).

### Analysis of N-soluble proteins, N-free amino acids and other N compounds in leaves

Soluble proteins were extracted from 200 mg of frozen fresh matter previously ground in a mortar with liquid nitrogen and 500 μL of citrate-phosphate buffer (20 mM citrate, 160 mM phosphate, pH 6.8 containing 50 mg of polyvinylpolypyrrolidone (PVPP)). After centrifugation (1 h, 12 000 *g*, 4°C), the resulting supernatant, containing the soluble proteins (protein extract, PE), was transferred to a microtube and completed to 500 μL. The concentration of the soluble proteins was determined in an equivalent of bovine serum albumin (BSA) using protein-dye staining [68]. To obtain the protein amount in equivalent nitrogen, an average N content of 16% was applied. For amino acid extraction, 1 mL of sodium phosphate 100 mM (pH 7.5) was added to 25 mg of leaf powder obtained after grinding of freeze-dried leaf samples. The mixture was incubated twice (30 min at 80°C) with 1 mL of 80% ethanol and 1 mL of 50% ethanol, respectively. After each incubation, the resulting mixtures were centrifuged (10 min, 12 000 *g*, 4°C) and the resulting supernatants containing amino acids were pooled, evaporated and re-suspended in 500 μL water. To quantify the amino acid concentration, 10 μL of amino acid extract were added to 90 μL of water and 1 mL of ninhydrin reagent (112 mM ninhydrin and 3.85 mM tin chloride in 100 mM citrate buffer pH 5, 50% (v/v) DMSO). After incubation for 20 min in boiling water and 10 min on ice, 5 mL of 50% ethanol were added and the absorbance was read at 570 nm. The amino acid concentration was calculated using L-leucine as standards. The N-amino acid amounts were deduced by using an average value for molecular mass (110 g.mol^−1^) and the N ratio (7.86) obtained from previous studies in leaf samples of oilseed rape was used to determine the N-amino acid amounts. In order to determine the amount of other N compounds (including insoluble proteins, oligopeptides, nitrate and ammonia), the N-soluble protein and N-amino acid amounts have been subtracted from the total N amount.

### Analysis of glutamate dehydrogenase (GDH) and glutamine synthetase (GS) in the source leaf

For GS and GDH analysis, proteins were extracted from 150 mg of fresh matter of leaves (previously ground in a mortar with liquid nitrogen) with 1 mL of extraction buffer (10 mM Na-EDTA, 10 mM MgCl_2_, 250 mM Tris–HCl pH 7.6, 13.3 mM β-mercaptoethanol and 2 mM leupeptine) containing 50 mg of polyvinylpyrrolidone (PVP). The GDH activity in the sense of glutamate synthesis (expressed in nmol of NAD(H) used.h^−1^.μg protein^−1^) was performed as previously described by Masclaux et *al.* [69]. The same protein extract was used to determine the GS activity (nmol glutamine.h^−1^.μg protein^−1^) by the method of O’Neal and Joy [70]. In order to monitor the kinetic evolution of GS1 and GS2, a western blot was performed. For this, the four biological replicates of protein extracts were pooled and 10 μg were denatured with Laemmli 2X buffer [71] containing β-mercaptoethanol (5% (v/v)). The proteins were separated on an SDS-PAGE gel (5.5% polyacrylamide (w/v) for the stacking gel and 10% polyacrylamide (w/v) for the resolving gel) and transferred to a polyvinylidene difluoride (PVDF) membrane. The protein detection of both GS (GS1 at 39 kDa and GS2 at 44 kDa) was made using rabbit polyclonal antibodies (1/100^e^) derived by Eurogenetec (Seraing, Belgium) against the peptide AYGENERRLTG. The antibodies were detected by secondary antibodies from goat (1/12 000^e^) coupled with alkaline phosphatase. The relative amount of GS1 (cytosolic isoform) and GS2 (chloroplastic isoform) was quantified by Image Lab software (Bio-Rad).

### Analysis of protease activities involved in Rubisco large subunit (LSU) degradation in the source leaf

In order to identify the proteases involved in the degradation of the Rubisco LSU, a new method using stain free gels (Bio-Rad®) was performed. These gels allow the detection of proteins immediately after the end of the SDS-PAGE and in less than 2 min, and the method is based on a UV-induced trihalo-compound modification of tryptophan residues contained in proteins (for details, see Kazmin et *al*. [72]). In order to identify the protease classes involved in the LSU degradation, the soluble protein extract (PE, 6 μg) obtained from source leaves at D7 was either incubated for 1 h at 37°C (t_1h_) or not (t_0_) in sodium acetate buffer 250 mM (pH 5, 0.16% β-mercaptoethanol (v/v)) with or without specific protease-class inhibitors. Iodoacetamide (14.5 mM) was used as an inhibitor of cysteine proteases (PE + CPI) and aprotinin (34 μM) was the serine proteases inhibitor (PE + SPI). For analysis of metalloprotease and aspartate protease activities, 5.5 mM of 1–10 phenanthroline (PE + Me + MI) and 10.2 μM of pepstatin A (PE + Me + API) were used, respectively. Finally, 40 μM of MG132 (benzyloxycarbonyl-leu-leu-leucinol) were used to inhibit proteasome (PE + DMSO + PI). Due to the solubility of 1–10 phenanthroline and pepstatin inhibitors in 1% methanol, the total protease activity was also quantified with 0.5 % methanol (v/v; PE + Me). In the same way, due to the solubility of MG132 in DMSO, the total protease activity was carried out with 0.5 % DMSO (PE + DMSO). At t_0_ or after incubation for 1 h at 37°C (t_1h_), Laemmli 2X buffer [71] containing β-mercaptoethanol (5% (v/v)) was added to the protein samples (one volume of Laemmli 2X buffer per volume of proteins) before protein denaturation for 7 min in boiling water. The resulting samples were loaded on SDS-PAGE (Mini-PROTEAN TGX Stain Free Gels 4-15%, Bio-Rad®) and the LSU amount at t_0_ (LSU_t0_) and after 1 h of incubation (LSU_t1h_) were visualized and quantified using Image Lab software (Bio-Rad®). The percentage of LSU degradation (% Deg) with or without protease inhibitors was firstly determined and used to deduce the inhibition of LSU proteolysis (% Inh) by the different protease inhibitors as follows:

$$ \%\ \mathrm{D}\mathrm{e}\mathrm{g} = \left({\mathrm{LSU}}_{\mathrm{t}0} - {\mathrm{LSU}}_{\mathrm{t}1\mathrm{h}}\right) \times \kern-0.35em 100\ /\ {\mathrm{LSU}}_{\mathrm{t}0} $$

$$ \%\mathrm{I}\mathrm{n}\mathrm{h} = \left({\mathrm{LSU}}_{\mathrm{PE}}\right)\ \hbox{--}\ \left({\mathrm{LSU}}_{\mathrm{PE}+\mathrm{I}\mathrm{n}\mathrm{h}}\right)\ *100\ /\ \left({\mathrm{LSU}}_{\mathrm{PE}}\right) $$

where LSU_PE_ is the amount of LSU degraded without inhibitor and LSU_PE+Inh_, is the amount of LSU degraded in the presence of protease inhibitors.

### Statistical analysis

The normality of the data was studied with the Ryan-Joiner test at 95%. Analysis of variance (ANOVA) and the Tukey test were used to compare the means. When the normality law of data was not respected, the non-parametric test of Kruskal-Wallis was carried out. Statistical significance was postulated at *P <* 0.05. Four biological repetitions corresponding to four different plants (*n* = 4) were used and the data presented here are expressed ± standard error (SE).

## Additional files

Additional file 1:
**Dry matter of every organs of Aviso and Oase in LN and HN conditions.** The data are expressed in grams. The plants were cultivated in ample (HN, 3.75 mM) or low (LN, 0.375 mM) nitrate concentrations and the data obtained at D0 (early bolting stage), D42 (pod formation), D70 (start of seed filling) and D99 (mature seed stage) are presented in this table. Grey box means that the organ was absent at this growing stage. Letters a, b, c represent the significant differences of organs between two harvest dates, the asterisks indicate significant differences between N treatments and hashes indicate differences between genotypes (*n* = 4 plants; *p* < 0.05). Additional file 2:
**N amount of every organs of Aviso and Oase in LN and HN conditions.** The data are expressed in milligrams. The plants were cultivated in ample (HN, 3.75 mM) or low (LN, 0.375 mM) nitrate concentrations and the data obtained at D0 (early bolting stage), D42 (pod formation), D70 (start of seed filling) and D99 (mature seed stage) are presented in this table. Grey box means that the organ was absent at this growing stage. Letters a, b, c represent the significant differences of organs between two harvest dates, the asterisks mean significant differences between N treatments and hashes mean differences between genotypes (*n* = 4 plants; *p* < 0.05). Additional file 3:
**N fluxes in Aviso (A) and Oase (B) in HN condition between D0 and D42.** The plants were supplied with high concentration of nitrate (HN, 0.375 mM of nitrate) and D0 corresponds to early bolting and D42 to pod formation. Fluxes of N from remobilization or uptake in the different organs are expressed as mg of N remobilized or taken up, respectively. For fluxes of N remobilisation, N amount is indicated with a minus sign (-) when N is remobilized from a source organ while it is indicated with a plus sign (+) when remobilized N is redistributed towards a sink organ. Asterisks represent significant differences between genotypes (*n* = 4 plants; *= *p* < 0.05). Additional file 4:
**N fluxes in Aviso (A) and Oase (B) in HN condition between D42 and D70.** The plants were supplied with high concentration of nitrate (HN, 0.375 mM of nitrate) and D42 corresponds to pod formation and D70 to the start of seed filling. Fluxes of N from remobilization or uptake in the different organs are expressed as mg of N remobilized or taken up, respectively. A shaded box means that the organ is not present during these growing stages. For fluxes of N remobilisation, N amount is indicated with a minus sign (-) when N is remobilized from a source organ while it is indicated with a plus sign (+) when remobilized N is redistributed towards a sink organ. Asterisks represent significant differences between genotypes (*n* = 4 plants; *= *p* < 0.05). Additional file 5:
**Description of the experimental design.** Two genotypes of winter oilseed rape (*Brassica napus* L. *cv.* Aviso and Oase) were cultivated in greenhouse conditions. After sterilization, seeds were sowed on watered vermiculite and when the first leaf appeared, plantlets were supplied during 2 months with a 25% Hoagland solution (for details see Material and Methods). Thereafter, plants were vernalized during 8 weeks and were transferred under greenhouse in pot containing perlite and vermiculite with a thermoperiod of 20°C (day) and 15°C (night). To estimate the N fluxes at whole plant level, a long term pulse chase labelling (at 2% atom excess) was applied from 43 days after sowing until early bolting growing stage (Day 0 of the experiment (D0) corresponding to GS31), including the vernalization period (8 weeks). At D0, ^15^N-NO_3_ labelling was stopped and two N treatments with unlabelled-NO_3_ were applied until the final harvest (D99; mature seeds *i.e.* GS99 stage): high nitrate (HN, 3.75 mM) and low nitrate (LN, 0.375 mM). Plants were harvested at different growing stage. Before the start of N limitation (D0), one mature leaf undergoing senescence (called “source leaf“, see picture showing the source leaf selected in Aviso plants) was studied in details for analysis of the physiological and molecular processes involved in leaf N remobilization. The “source leaf” was selected on the following criteria: leaf that attained its maximal expansion (leaf area), with a high chlorophyll content and having the same position in the canopy in both genotypes. The leaf biomass at D0 (3.03 ± 0.06 g FW for Aviso vs 2.77 ± 0.13 g FW for Oase), leaf area from D0 to D28 (103.57 ± 4.71 cm^2^ for Aviso vs 99.45 ± 3.89 cm^2^ for Oase) and chlorophyll content at D0 (SPAD value of 51.25 ± 0.71 for Aviso vs 52.53 ± 1.28 for Oase) in source leaves were not significantly different between Aviso and Oase. Additional file 6:
**Number and residual N in dead leaves of Aviso and Oase.** The plants were cultivated in ample (HN, 3.75 mM) or low (LN, 0.375 mM) nitrate concentrations and the data obtained at D0 (early bolting stage), D42 (pod formation), D70 (start of seed filling) and D99 (mature seed stage) are presented in this table. Asterisks represent differences between N treatments and hashes differences between genotypes (*n* = 4 plants; *= *p* < 0.05). Additional file 7:
**Rubisco large subunit degradation in a selected source leaf with or without protease inhibitors.** The Rubisco large subunit (LSU) in the soluble protein extract (PE) of the source leaf (7 days after bolting) is visualized on stain free SDS-PAGE and quantified for the four biological repetitions (A, B, C and D) by Image Lab software (Bio-Rad) at (t0) and after 1 h of incubation at 37°C (t1h) without inhibitors (PE) or with iodoacetamide (PE + CPI; cystein protease inhibitor), aprotinin (PE + SPI; serine protease inhibitor), methanol (PE + Me), methanol and 1-10 phenanthroline (PE + Me + MI; metalloprotease inhibitor), methanol and pepstatin A (PE + Me + API; aspartic protease inhibitor), DMSO (PE + DMSO) or DMSO and MG132 (PE + DMSO + PI; proteasome inhibitor). The percentage of degradation is indicated below the gel (in red). The mean of the percentage of degradation of the 4 biological repetitions ± standard error is indicated in the table on the right of the gel.

## Abbreviations

N: Nitrogen
HN: High nitrogen
LN: Low nitrogen
DM: Dry matter
TSW: Thousand seed weight
LSU: Rubisco large subunit
GS: Glutamine synthetase
GDH: Glutamate dehydrogenase
NUE: Nitrogen use efficiency
NUtE: Nitrogen utilization efficiency
NAE: Nitrogen assimilation efficiency
NRE: Nitrogen remobilization efficiency
NUpE: Nitrogen uptake efficiency
FtsH: Filamentation temperature-sensitive H
