# Supplementary figures and images for: The contrasting N management of two oilseed rape genotypes reveals the mechanisms of proteolysis associated with leaf N remobilization and the respective contributions of leaves and stems to N storage and remobilization during seed filling
Source: BMC Plant Biol. 2015 Feb 21;15:59. doi: 10.1186/s12870-015-0437-1 (PMC4384392; doi:10.1186/s12870-015-0437-1)

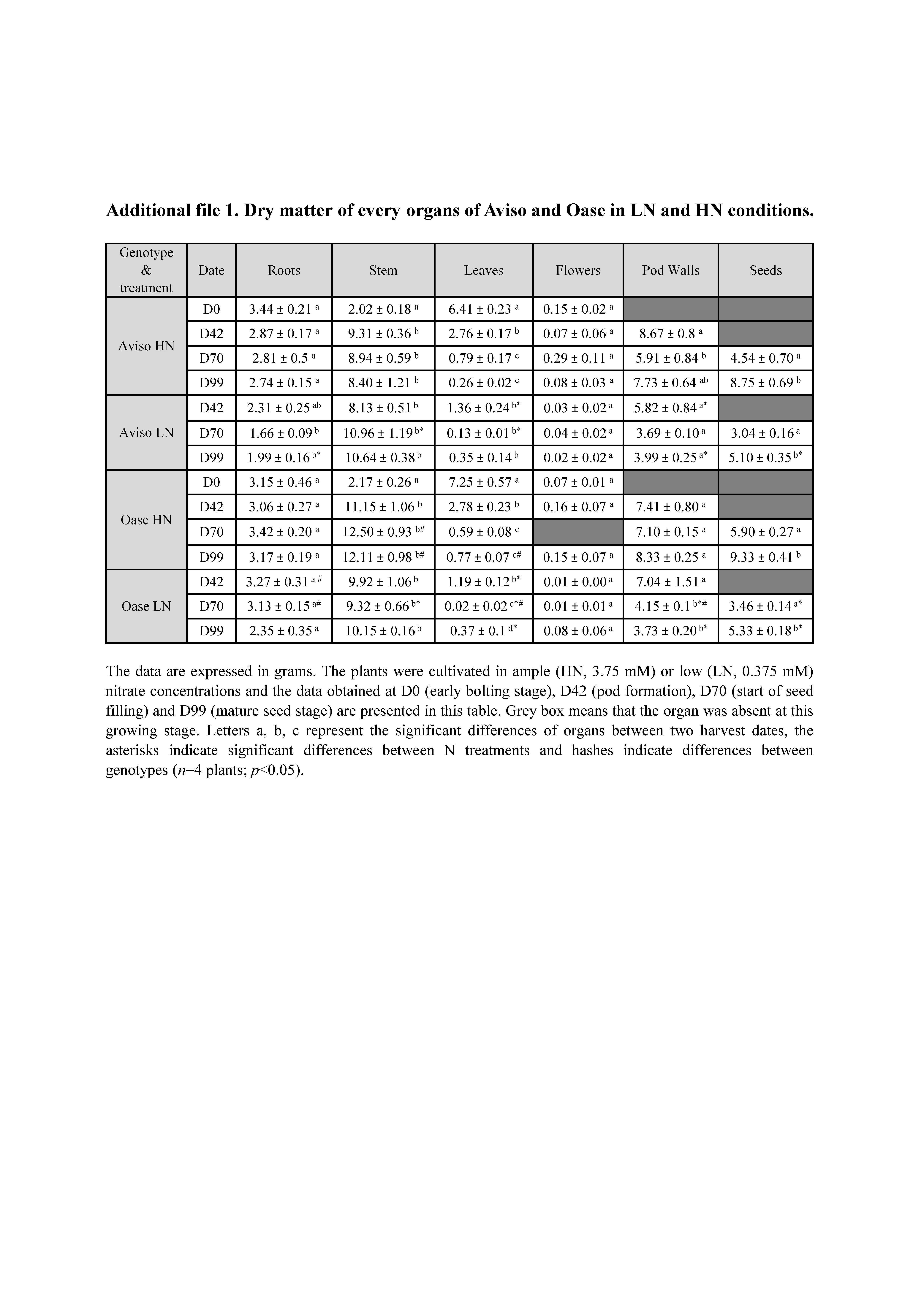

Supplement: Additional file 1: — Dry matter of every organs of Aviso and Oase in LN and HN conditions. The data are expressed in grams. The plants were cultivated in ample (HN, 3.75 mM) or low (LN, 0.375 mM) nitrate concentrations and the data obtained at D0 (early bolting stage), D42 (pod formation), D70 (start of seed filling) and D99 (mature seed stage) are presented in this table. Grey box means that the organ was absent at this growing stage. Letters a, b, c represent the significant differences of organs between two harvest dates, the asterisks indicate significant differences between N treatments and hashes indicate differences between genotypes (n = 4 plants; p < 0.05). [file 12870_2015_437_MOESM1_ESM.tiff]

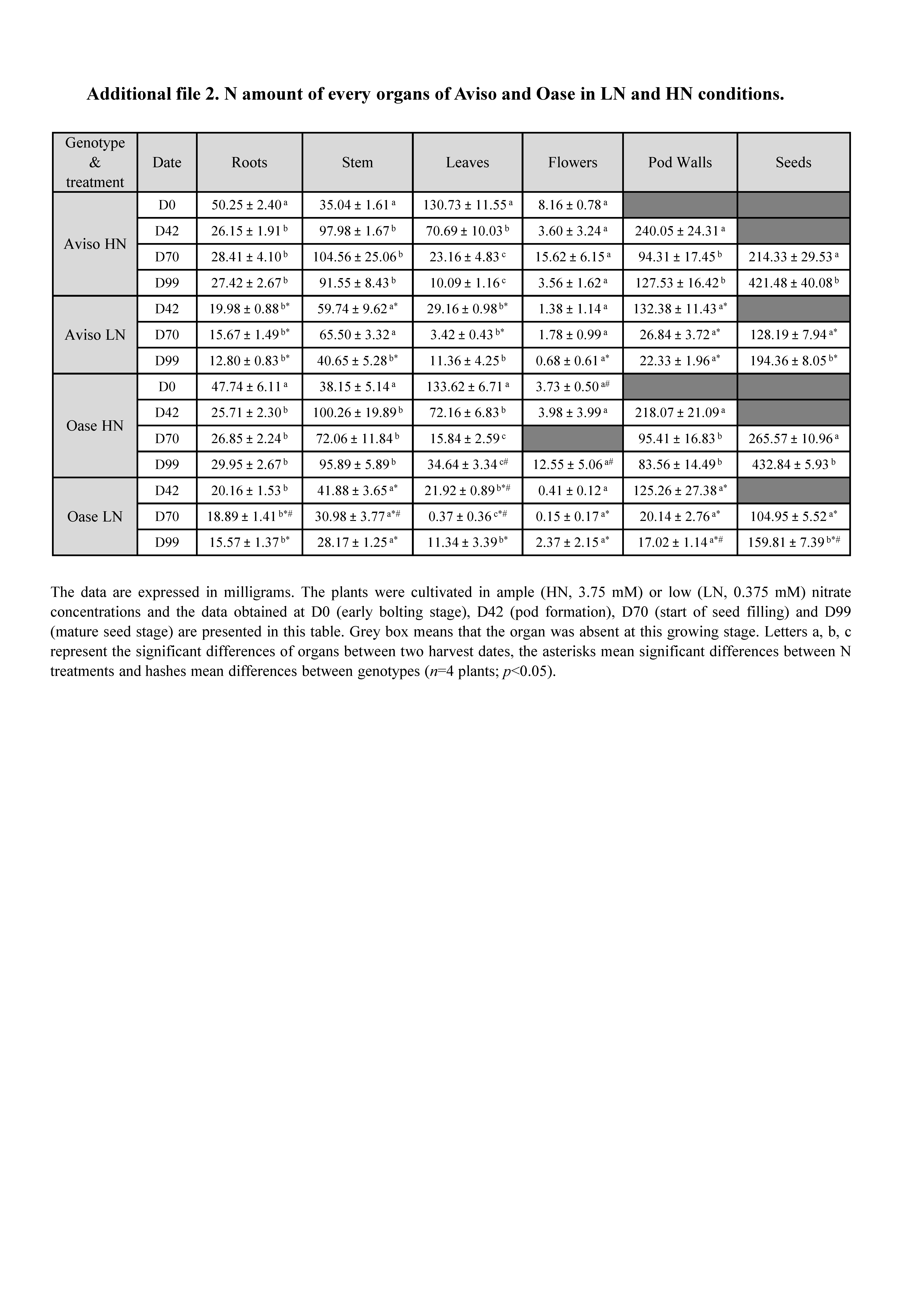

Supplement: Additional file 2: — N amount of every organs of Aviso and Oase in LN and HN conditions. The data are expressed in milligrams. The plants were cultivated in ample (HN, 3.75 mM) or low (LN, 0.375 mM) nitrate concentrations and the data obtained at D0 (early bolting stage), D42 (pod formation), D70 (start of seed filling) and D99 (mature seed stage) are presented in this table. Grey box means that the organ was absent at this growing stage. Letters a, b, c represent the significant differences of organs between two harvest dates, the asterisks mean significant differences between N treatments and hashes mean differences between genotypes (n = 4 plants; p < 0.05). [file 12870_2015_437_MOESM2_ESM.tiff]

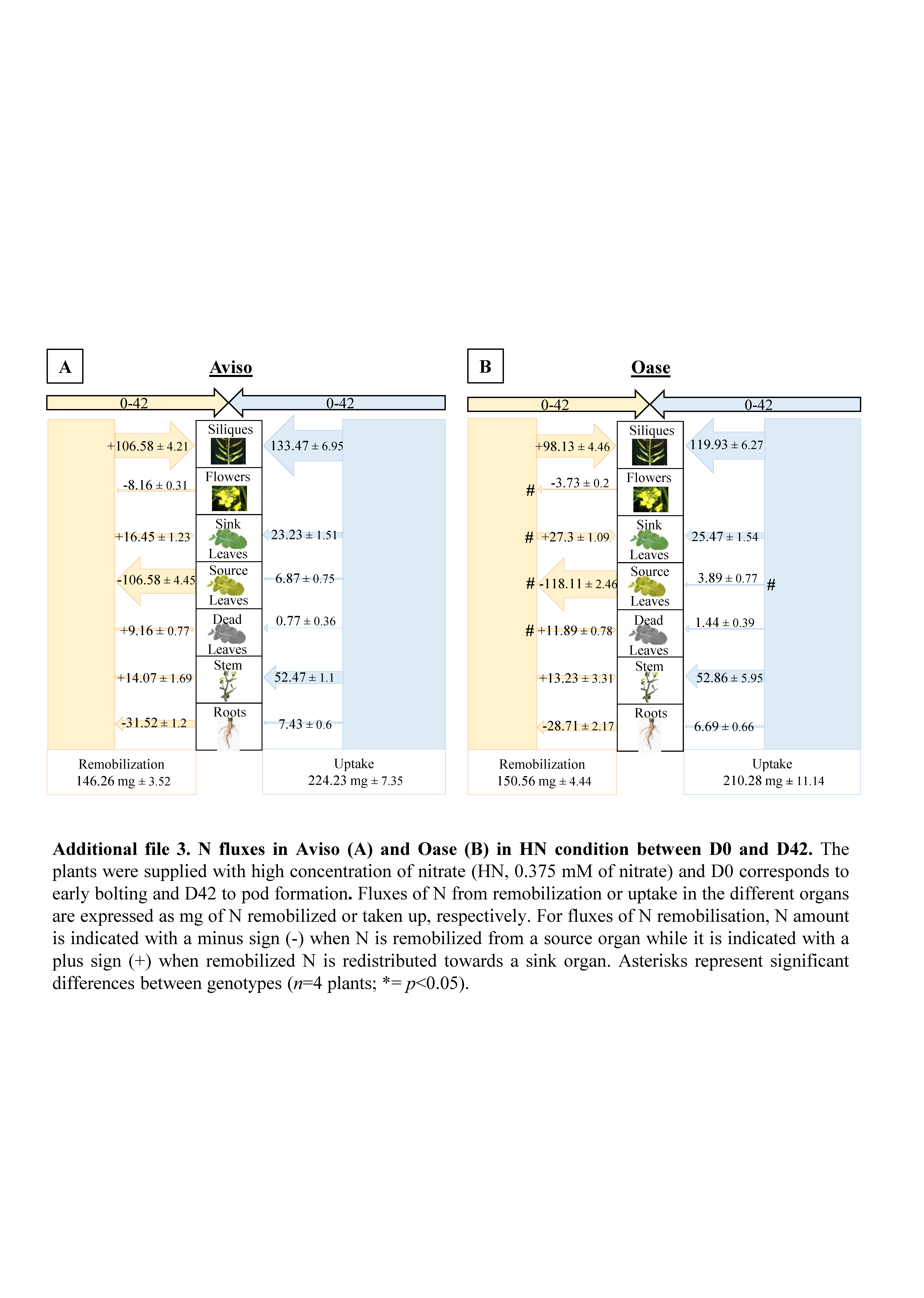

Supplement: Additional file 3: — N fluxes in Aviso (A) and Oase (B) in HN condition between D0 and D42. The plants were supplied with high concentration of nitrate (HN, 0.375 mM of nitrate) and D0 corresponds to early bolting and D42 to pod formation. Fluxes of N from remobilization or uptake in the different organs are expressed as mg of N remobilized or taken up, respectively. For fluxes of N remobilisation, N amount is indicated with a minus sign (-) when N is remobilized from a source organ while it is indicated with a plus sign (+) when remobilized N is redistributed towards a sink organ. Asterisks represent significant differences between genotypes (n = 4 plants; *= p < 0.05). [file 12870_2015_437_MOESM3_ESM.tiff]

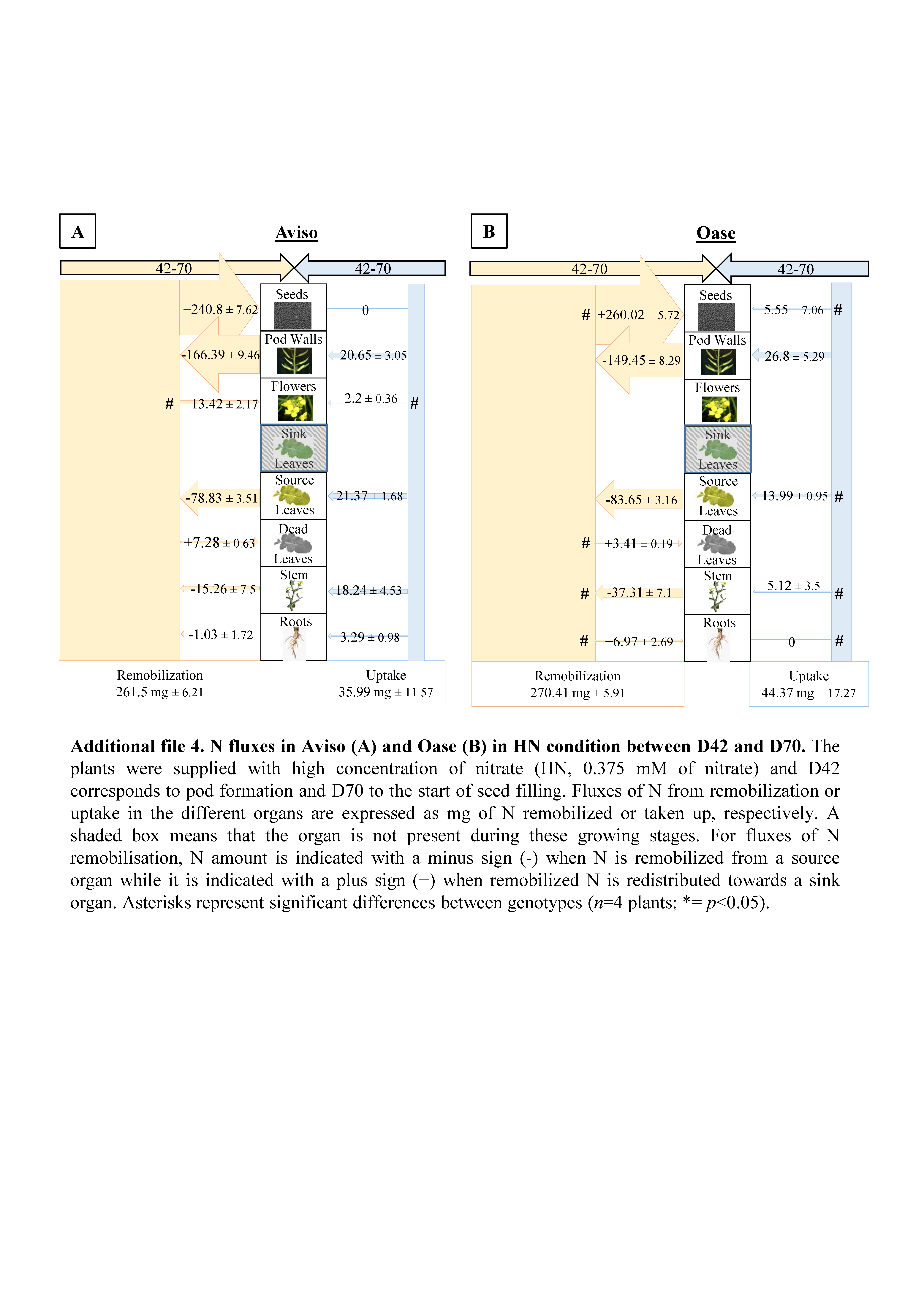

Supplement: Additional file 4: — N fluxes in Aviso (A) and Oase (B) in HN condition between D42 and D70. The plants were supplied with high concentration of nitrate (HN, 0.375 mM of nitrate) and D42 corresponds to pod formation and D70 to the start of seed filling. Fluxes of N from remobilization or uptake in the different organs are expressed as mg of N remobilized or taken up, respectively. A shaded box means that the organ is not present during these growing stages. For fluxes of N remobilisation, N amount is indicated with a minus sign (-) when N is remobilized from a source organ while it is indicated with a plus sign (+) when remobilized N is redistributed towards a sink organ. Asterisks represent significant differences between genotypes (n = 4 plants; *= p < 0.05). [file 12870_2015_437_MOESM4_ESM.tiff]

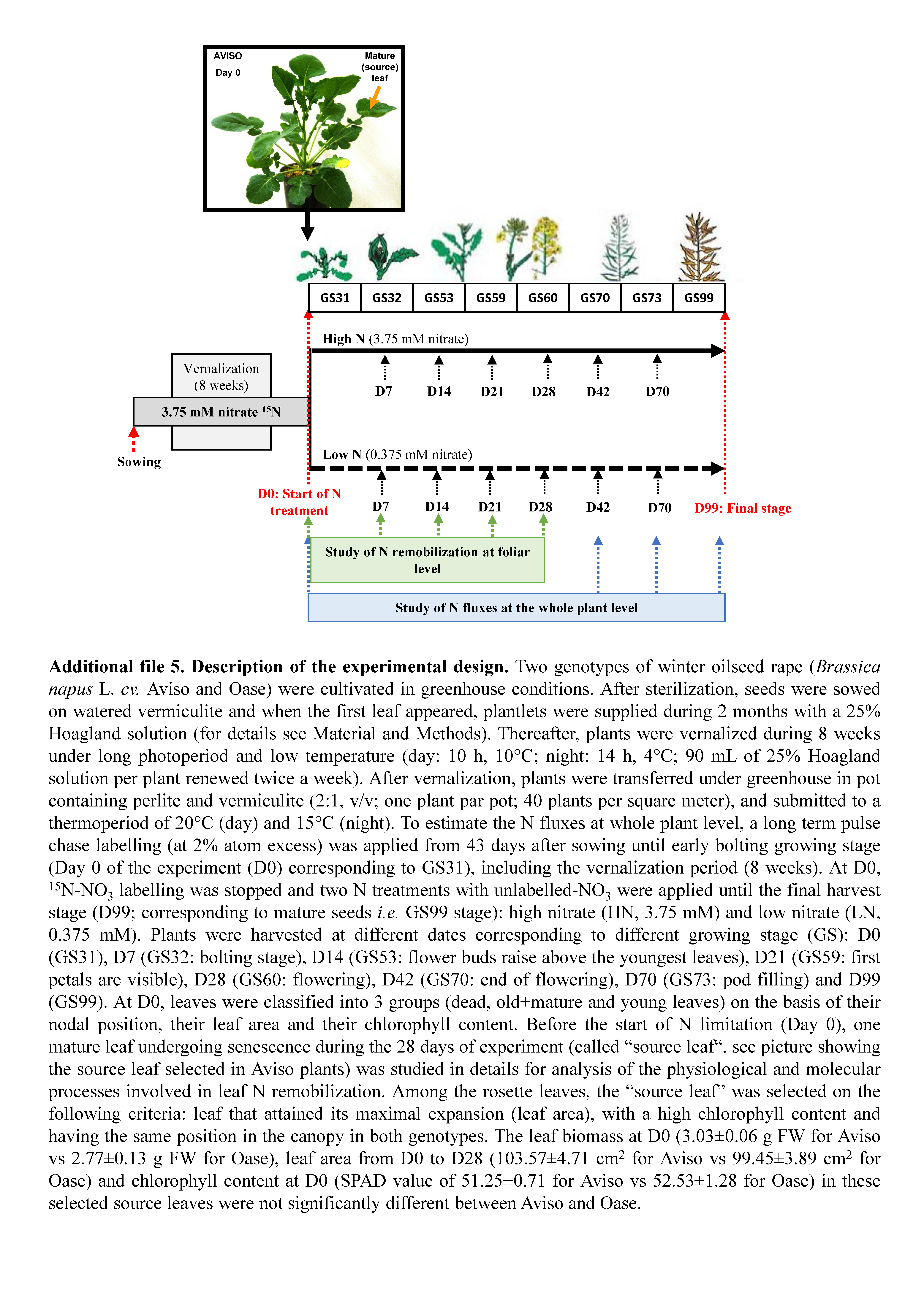

Supplement: Additional file 5: — Description of the experimental design. Two genotypes of winter oilseed rape (Brassica napus L. cv. Aviso and Oase) were cultivated in greenhouse conditions. After sterilization, seeds were sowed on watered vermiculite and when the first leaf appeared, plantlets were supplied during 2 months with a 25% Hoagland solution (for details see Material and Methods). Thereafter, plants were vernalized during 8 weeks and were transferred under greenhouse in pot containing perlite and vermiculite with a thermoperiod of 20°C (day) and 15°C (night). To estimate the N fluxes at whole plant level, a long term pulse chase labelling (at 2% atom excess) was applied from 43 days after sowing until early bolting growing stage (Day 0 of the experiment (D0) corresponding to GS31), including the vernalization period (8 weeks). At D0, 15N-NO3 labelling was stopped and two N treatments with unlabelled-NO3 were applied until the final harvest (D99; mature seeds i.e. GS99 stage): high nitrate (HN, 3.75 mM) and low nitrate (LN, 0.375 mM). Plants were harvested at different growing stage. Before the start of N limitation (D0), one mature leaf undergoing senescence (called “source leaf“, see picture showing the source leaf selected in Aviso plants) was studied in details for analysis of the physiological and molecular processes involved in leaf N remobilization. The “source leaf” was selected on the following criteria: leaf that attained its maximal expansion (leaf area), with a high chlorophyll content and having the same position in the canopy in both genotypes. The leaf biomass at D0 (3.03 ± 0.06 g FW for Aviso vs 2.77 ± 0.13 g FW for Oase), leaf area from D0 to D28 (103.57 ± 4.71 cm2 for Aviso vs 99.45 ± 3.89 cm2 for Oase) and chlorophyll content at D0 (SPAD value of 51.25 ± 0.71 for Aviso vs 52.53 ± 1.28 for Oase) in source leaves were not significantly different between Aviso and Oase. [file 12870_2015_437_MOESM5_ESM.tiff]

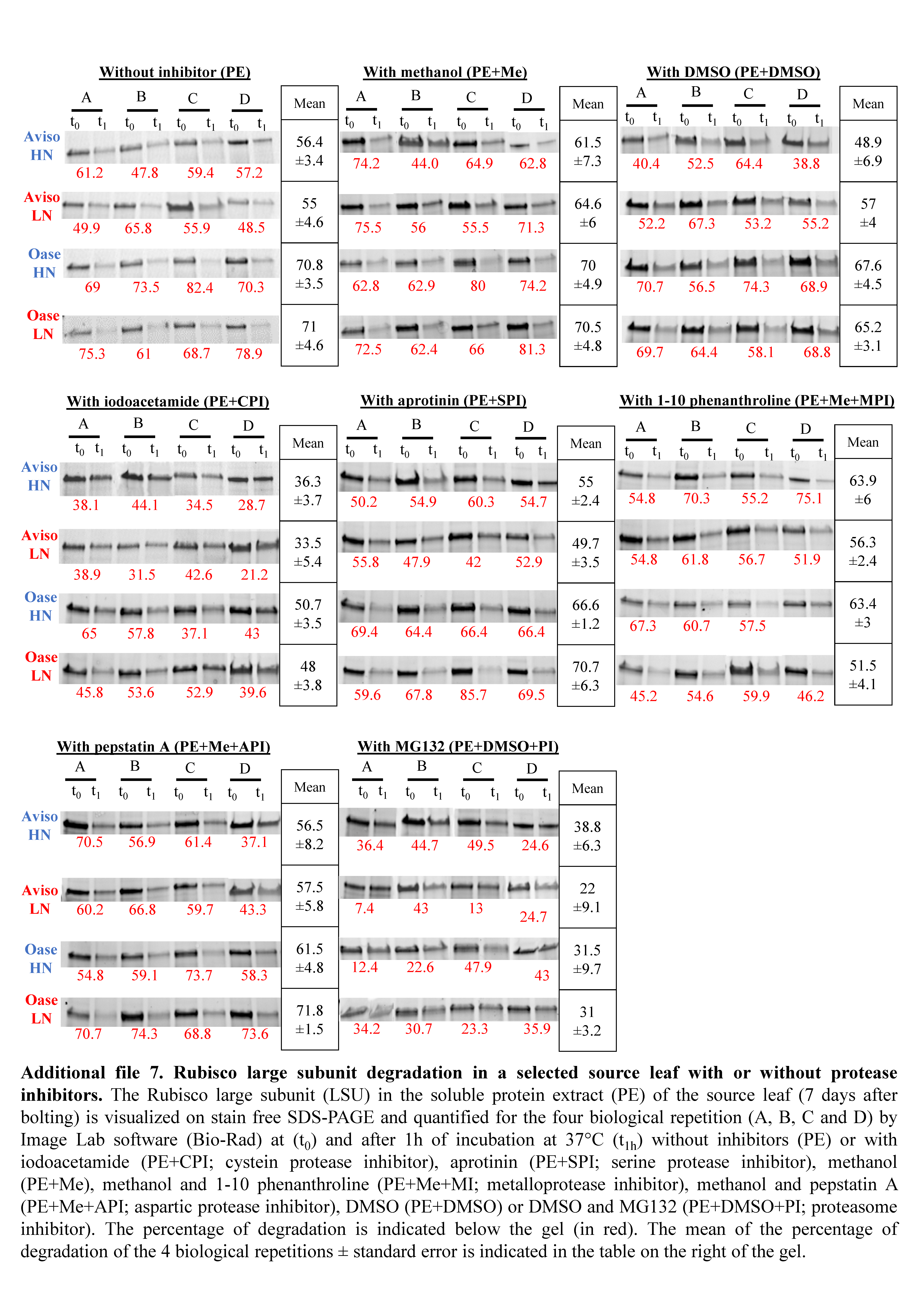

Supplement: Additional file 7: — Rubisco large subunit degradation in a selected source leaf with or without protease inhibitors. The Rubisco large subunit (LSU) in the soluble protein extract (PE) of the source leaf (7 days after bolting) is visualized on stain free SDS-PAGE and quantified for the four biological repetitions (A, B, C and D) by Image Lab software (Bio-Rad) at (t0) and after 1 h of incubation at 37°C (t1h) without inhibitors (PE) or with iodoacetamide (PE + CPI; cystein protease inhibitor), aprotinin (PE + SPI; serine protease inhibitor), methanol (PE + Me), methanol and 1-10 phenanthroline (PE + Me + MI; metalloprotease inhibitor), methanol and pepstatin A (PE + Me + API; aspartic protease inhibitor), DMSO (PE + DMSO) or DMSO and MG132 (PE + DMSO + PI; proteasome inhibitor). The percentage of degradation is indicated below the gel (in red). The mean of the percentage of degradation of the 4 biological repetitions ± standard error is indicated in the table on the right of the gel. [file 12870_2015_437_MOESM7_ESM.tiff]
